# Geographic and Social Equity in Population-Wide Genomic Screening

**DOI:** 10.1001/jamanetworkopen.2026.22743

**Published:** 2026-07-13

**Authors:** Kalyani Sonawane, Daniel P. Judge, Ella Moore, Samantha Norman, Caitlin G. Allen

**Affiliations:** 1 Department of Public Health Sciences, College of Medicine, Medical University of South Carolina, Charleston; 2 Hollings Cancer Center, Medical University of South Carolina, Charleston; 3 Department of Medicine, College of Medicine, Medical University of South Carolina, Charleston; 4 Department of Genetics and Genomics, Medical University of South Carolina, Charleston; 5 Department of Public Health Sciences, Wake Forest University School of Medicine, Winston-Salem, North Carolina

## Abstract

**Importance:**

Genomic screening for Centers for Disease Control and Prevention Tier 1 conditions, ie, hereditary breast and ovarian cancer syndrome (HBOC), Lynch syndrome, and familial hypercholesterolemia, enables early detection of these rare, but highly penetrant disorders and supports timely, evidence-based interventions. However, states with large rural and socially disadvantaged populations, such as South Carolina, face substantial structural and access-related barriers that limit the reach of population-wide genomic screening (PWGS).

**Objective:**

To examine whether combining implementation science and informatics infrastructure supports PWGS and to assess screening coverage and positivity by rurality and social disadvantage.

**Design, Setting, and Participants:**

This cross-sectional study used data from In Our DNA SC, a statewide PWGS program launched on November 8, 2021, in South Carolina. Recruitment and sample collection occurred via the health system, community-based collection events, and at-home kits. Analyses included adults aged 18 or older who completed screening as of July 23, 2025.

**Exposure:**

Genomic screening for Centers for Disease Control and Prevention Tier 1 conditions.

**Main Outcomes and Measures:**

County-level screening coverage rates (per 100 000 population) and positivity prevalence (per 100 000 valid results) were stratified using rural-urban continuum (RUC) codes (1 = most urban to 9 = most rural) and Social Vulnerability Index (SVI) quartiles (from 1 = least disadvantaged to 4 = most disadvantaged).

**Results:**

Of a total of 82 420 adults enrolled in the PWGS program, 50 897 completed screening (71.6% aged <65 years and 28.4% aged ≥65 years, 72.6% female). The program enrolled and screened participants from all 46 South Carolina counties. Screening coverage rates varied by RUC code and SVI. Screening rates per 100 000 population exceeded 1000 in RUC codes 1 through 3 and were significantly higher than RUC code 6 (791.4; 95% CI, 757.1-827.2), RUC code 8 (861.4; 95% CI, 801.1-926.2), and RUC code 9 (839.6; 95% CI, 683.6-1030.8). Screening coverage by overall SVI score was significantly higher in quartile 1 (1422.1; 95% CI, 1406.3-1438.1) and quartile 2 (1188.4; 95% CI, 1167.3-1209.9) compared with quartile 3 (888.4; 95% CI, 866.5-910.9) and quartile 4 (839.6; 95% CI, 952.3-1025.4). Positivity prevalence was generally similar across RUC codes, except for HBOC, which was significantly higher per 100 000 in RUC code 2 (801.3; 95% CI, 715.3-897.4) compared with RUC code 1 (268.5; 95% CI, 123.1-584.5). When examined by overall SVI quartiles, positivity prevalence for HBOC, Lynch syndrome, and familial hypercholesterolemia remained consistent, with no statistically significant differences across levels of disadvantage.

**Conclusions and Relevance:**

This cross-sectional study of a statewide PWGS program supported by an implementation framework and informatics platform suggests broad geographic and social reach. Evaluation of clinical and behavioral outcomes is the critical next step for determining program effectiveness and performance in community and clinical settings. Taken together, these findings suggest that pairing implementation science with robust informatics infrastructure may support PWGS delivery in diverse communities.

## Key Points

**Question:**

Does the integration of implementation science and informatics infrastructure support equitable access to population-wide genomic screening (PWGS) across diverse geographic and socially disadvantaged communities?

**Findings:**

In this cross-sectional study of 50 897 adults enrolled in a PWGS program in South Carolina who completed screening for hereditary breast and ovarian cancer, Lynch syndrome, and familial hypercholesterolemia, screening coverage (per 100 000 population) ranged from 700 to more than 880 across all levels of rurality and social disadvantage, respectively. Test positivity varied by rurality and social disadvantage.

**Meaning:**

These findings suggest that combining implementation science with informatics infrastructure may support scalable and equitable PWGS delivery.

## Teaser

This cross-sectional study reports on the implementation of a population-wide genomic screening program in South Carolina and assesses rates of screening for genetic disorders and positivity prevalence by rurality and social disadvantage.

## Introduction

The National Academy of Medicine Genomics identifies hereditary breast and ovarian cancer syndrome (HBOC), Lynch syndrome, and familial hypercholesterolemia as Centers for Disease Control and Prevention (CDC) Tier 1 applications for genomic screening. Detection of these rare, but highly penetrant conditions enables evidence-based interventions that can substantially improve outcomes for individuals with these conditions and their families.^1^ Although most cardiovascular diseases and cancers arise from multifactorial and polygenic causes, access to screening for these monogenic conditions remains uneven and mirrors broader inequities in health care delivery. States with large rural and socially disadvantaged populations, such as South Carolina, face disproportionate burdens of cardiovascular disease and cancer, conditions for which early genomic risk identification could be transformative. The state of South Carolina has striking demographic and geographic diversity, with more than 5.5 million residents spread across urban centers, small towns, and extensive rural communities.^2^ Nearly one-quarter of the population identifies as Black or African American, and many counties rank high on measures of social disadvantage due to poverty, limited health care access, and transportation barriers.^3^ Expanding access to CDC Tier 1 genomic screening in this context represents a critical opportunity to advance health equity by ensuring that individuals and families who stand to benefit from preventive interventions are identified and supported.

Recognizing the role of genetics in chronic disease prevention and management, South Carolina’s Medicaid system implemented a landmark policy in 2019 that covers *BRCA* genetic testing for eligible men and women who meet 1 or more of the genetic or familial high-risk assessment criteria for breast and ovarian cancer.^4,5^ More recently, coverage has expanded to include testing for whole-exome sequencing, whole-genome sequencing, and hereditary cancer panels when clinically indicated.^6^ These policy advances signal the state’s commitment to precision medicine, yet use of genetic testing remains low, especially among rural and underserved populations. To maximize population impact, establishing a population-wide genomic screening (PWGS) program that ensures equitable access to and successful implementation of genetic screening for all individuals across the state was necessary.

To address the need for equitable access to genomic screening, the Medical University of South Carolina (MUSC) launched In Our DNA SC in 2021, intending to enroll at least 100 000 participants to receive no-cost genomic screening for CDC Tier 1 conditions.^7^ The program adopted an innovative PWGS model by integrating implementation science strategies to evaluate the program.^7^ In parallel, it established a robust informatics infrastructure, including a web-based data visualization platform, to monitor and communicate program enrollment and screening completion.^8^ Ensuring representation across all counties, spanning the rural-urban continuum (RUC) and communities with varying levels of social disadvantage, was a central priority in building a cohort representative of the state’s diverse population.

The objectives of this study included plans to (1) describe the strategic implementation agenda for South Carolina’s PWGS program (In Our DNA SC), (2) examine how informatics infrastructure could support monitoring the PWGS program, and (3) descriptively assess equity in screening coverage by rurality and social disadvantage to evaluate program success in engaging diverse communities across South Carolina.

## Methods

### Setting

This cross-sectional study examined data from the In Our DNA SC research project launched on November 8, 2021, by MUSC. The no-cost genetic screening program is an academic-private partnership run by MUSC in collaboration with Helix. The In Our DNA SC protocol was approved by the WIRB-Copernicus Group. The analyses used deidentified data and were deemed exempt from full review by the MUSC Institutional Review Board. Participants provided electronic or written informed consent. This study followed the Strengthening the Reporting of Observational Studies in Epidemiology (STROBE) reporting guideline for cross-sectional studies.

### Participant Recruitment and Genetic Screening

Participants enrolled electronically using their MUSC electronic health record credentials and were able to provide their biospecimen through 2 pathways: (1) in-person collection completed during a clinical appointment at a participating MUSC clinic, a participating MUSC laboratory, or a community-based collection event or (2) at-home collection. Demographic information, including age, race (Black, White, or other [American Indian or Alaska Native, Asian Indian, Chinese, Filipino, Guamanian or Chamorro, Native Hawaiian, Samoan, Japanese, Other Asian, Vietnamese, or multiracial, collapsed due to small numbers]), and ethnicity (Hispanic or Latino, not Hispanic or Latino, or unknown) was self-reported by participants. Race and ethnicity were included to ensure representation of all racial and ethnic groups. Eligible individuals were recruited via the electronic health record ahead of their upcoming clinic appointment. After completing electronic informed consent, the platform generated the necessary order, and participants provided their specimen during their clinic visit or at a participating laboratory.

Community-based collection events were conducted across South Carolina in partnership with local colleges, businesses, and community organizations to enhance reach and broaden participation. At these events, participants completed the consent form and provided a sample, assisted by trained study staff. For at-home collection, participants were mailed a test kit with detailed instructions for sample collection and were asked to return the completed kit to Helix using a prepaid shipping label. Samples were sequenced by Helix for the CDC Tier 1 conditions in a Clinical Laboratory Improvement Amendments–certified laboratory, including the evaluation of *BRCA1*, *BRCA2*, *MLH1*, *MSH2*, *MSH6*, *PMS2*, *EPCAM*, *APOB*, *LDLR*, *LDLRAP1*, and *PCSK9*.

### Genetic Screening Results and Counseling

Participants received their CDC Tier 1 condition results through their electronic health record portal approximately 8 to 12 weeks after sample collection. Negative results were released automatically, while participants with a pathogenic or likely pathogenic (P/LP) variant for 1 of the hereditary conditions were contacted by study staff for results disclosure. If participants could not be reached, results were communicated via certified letter. Participants with a P/LP variant were offered a free genetic counseling appointment to review results, discuss follow-up care, and access condition-specific resources, including colonoscopy among individuals with Lynch syndrome, breast imaging among those with HBOC-associated variants, and treatment initiation for individuals with familial hypercholesterolemia. Individuals who declined counseling received a gene guide with lifestyle recommendations, medical management information, and support group resources. Additional details of the program and processes are available elsewhere.^7^ All results were included in the participant’s medical records and available for their clinical team to review.

### Implementation Framework

The study team developed an evaluation plan guided by the reach, effectiveness, adoption, implementation, and maintenance (RE-AIM) implementation science framework, which assesses factors that influence the translation of innovations at both individual and organizational levels.^9^ The eFigure in Supplement 1 provides an overview of how the RE-AIM framework was adapted for the In Our DNA SC program.^7^ The study team identified metrics for measurement and evaluation as follows: reach, the number of individuals reached and participants enrolled; effectiveness, number of participants completing the program, identification of P/LP variants for CDC Tier 1 conditions, and uptake of genetic counseling referrals; adoption, the number of opportunities to enroll across clinics, community events, and at-home options; implementation, program delivery through adaptations at the site level and sample collection, recollection, and timeliness of results at the individual level; and maintenance, the sustainability of enrollment opportunities and the ongoing clinical management of high-risk individuals identified with P/LP variants.

### Informatics Infrastructure

To monitor program reach and effectiveness, including the geographic scope and representativeness of individuals from rural and socially disadvantaged areas, program data were integrated into a broader institution-wide resource, the South Carolina–Cancer Surveillance for Population Health Research and Outreach Tool, an interactive catchment area data visualization dashboard.^10^ The dashboard provides summary metrics on enrolled participants (reach), completed screenings (effectiveness), and P/LP results (effectiveness). This informatics resource was developed by researchers at MUSC using a stakeholder-driven approach guided by a health communications framework. Researchers and community stakeholders worked collaboratively to develop a data visualization dashboard, which was tested using a mixed-methods approach to ensure that population health data (eg, new cases of diseases, deaths, risk factors, social determinants of health) are effectively communicated. Deidentified data were aggregated to the county level and geolinked with rural-urban classifications and social disadvantage measures.

Rural-urban continuum codes were derived from the US Department of Agriculture’s Economic Research Service, while county social disadvantage scores were obtained from the CDC’s Social Vulnerability Index (SVI). For each county, the RUC code and SVI quartile were identified. The RUC codes classify counties into 9 categories, from 1 (most urban) to 9 (most rural), based on metropolitan population size, degree of urbanization, and proximity to metropolitan areas. They distinguish metropolitan counties by the size of their metropolitan area and nonmetropolitan counties by how urbanized they are and whether they are adjacent to a metropolitan area. The SVI measures how well communities can prepare for, respond to, and recover from disasters by assessing social and demographic factors. It combines multiple characteristics into 4 themes: socioeconomic status (eg, poverty, unemployment, income, education), household composition and disability (eg, age, disability, single-parent households), minority status and language (eg, race and ethnicity, English proficiency), and housing type and transportation (eg, crowded housing, access to vehicles). Each community receives a score from 0 (least disadvantaged) to 1 (most disadvantaged), helping identify areas needing targeted support.

### Statistical Analysis

Data were analyzed based on data collected as of July 23, 2025. Frequency distributions were used to describe the number of participants who enrolled, were screened, and had positive (P/LP) results overall, in each county, and by rurality and SVI categories. Proportions were calculated for participants who completed screening (of total enrolled) and for those who received positive (P/LP) results (of the total with valid test results). Geographic and social equity in screening coverage, defined as screening rates per 100 000 adults aged 18 years or older, was evaluated by estimating for each of the 46 South Carolina counties and for RUC and SVI groupings. Positivity prevalence, defined as the number of P/LP findings per 100 000 valid test results, was calculated for counties and for RUC and SVI groupings with at least 3 valid results to allow meaningful comparisons by rurality and social disadvantage. Wilson confidence intervals were generated for both screening rates and positivity prevalence. Standardized scores were used to compare participant characteristics with the state population. A multiple variable logistic regression model adjusted for age, sex, race, and ethnicity was used to examine the likelihood of withdrawal after initial consent. Statistical significance was assessed at *P* < .05. All analyses were performed using SAS, version 9.4 (SAS Institute, Inc).

County-level data were converted into a format readable in the Tableau, version 2025.2 visualization software (Tableau Software, Inc). All visualizations were developed and rendered in the Tableau environment. The data visualization dashboards were embedded on the MUSC public-facing website. Interactivity is maintained using the Tableau JavaScript application program interface. All data visuals are downloadable in commonly used formats (image, presentation, and spreadsheet) to facilitate dissemination. A detailed description of the informatics resource development process is available elsewhere.^8^

## Results

### Participant Characteristics

A total of 82 420 participants had enrolled as of July 23, 2025, representing individuals from all 46 counties in South Carolina; 542 withdrew before screening. By July 2025, 50 897 participants (61.8%) had completed screening (71.6% aged <65 years and 28.4% aged ≥65 years; 72.6% female and 27.4% male; 9.3% self-identifying as Black, 73.1% as White, and 17.6% as other race; 2.5% self-identifying as Hispanic or Latino, 81.2% as not Hispanic or Latino, and 16.3% as unknown ethnicity) (eTable 1 in Supplement 1). Modest differences were observed by sex and race compared with the state population. The variants identified among screened participants with P/LP results are listed in eTable 2 in Supplement 1.

### County-Level Characteristics, Enrollment, Screening Coverage, and Test Positivity

**Figure 1.**
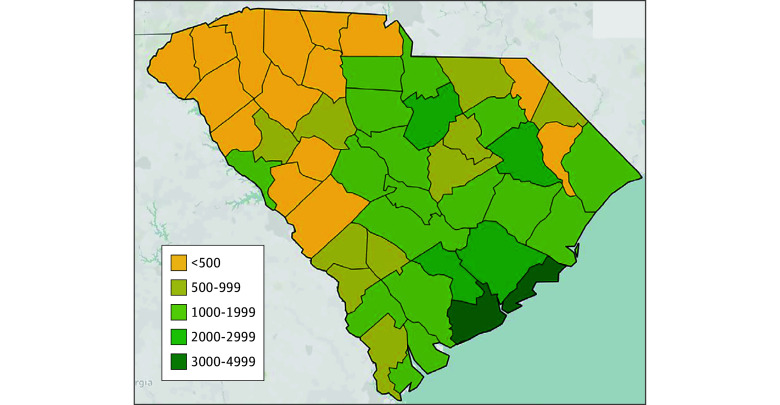
Map of Genetic Screening Coverage Rates by County, In Our DNA SC Program Rates are per 100 000 across the 46 South Carolina counties. Participants who consented and enrolled in the In Our DNA SC program received genetic screening for Centers for Disease Control and Prevention Tier 1 conditions.

Characteristics of the 46 South Carolina counties are described in eTable 3 in Supplement 1. Charleston County had the highest enrollment (5880 per 100 000 population) and accounted for the largest share of screened participants (12 500 [24.6%]).^10^ Per 100 000 population, the screening coverage rate was significantly higher in Charleston County (3751.4; 95% CI, 3687.4-3816.4) compared with all other counties (Figure 1). The screening coverage rate was significantly lower per 100 000 in Cherokee County (142.9; 95% CI, 111.5-183.2) compared with Charleston County.

**Figure 2.**
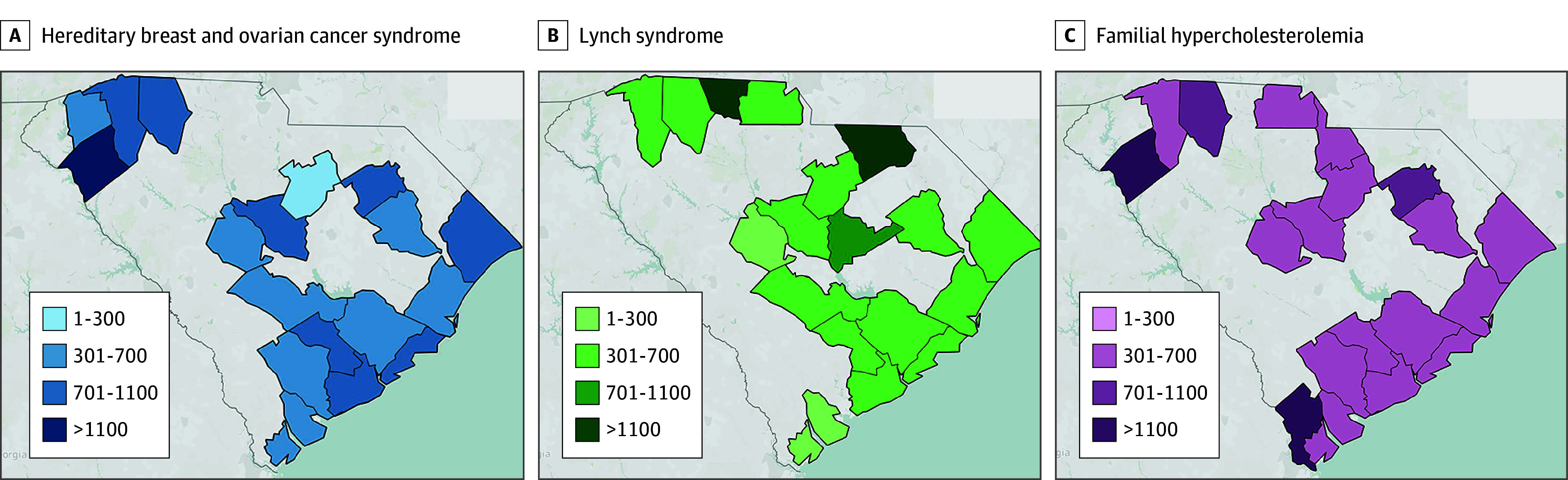
Maps of Positive Test Results by County, In Our DNA SC Program Positivity prevalence per 100 000 tests across South Carolina for counties with a minimum of 3 positive test findings. Participants who consented and were enrolled in the In Our DNA SC program received genetic screening for Centers for Disease Control and Prevention Tier 1 conditions. Positive results included likely pathogenic or pathogenic findings, and positivity prevalence was calculated after excluding invalid results.

Positivity prevalence per 100 000 valid tests for counties with a minimum of 3 positive results was higher for HBOC in Anderson (2229.0; 95% CI, 1409.9-3789.8) vs Charleston (848; 95% CI, 714.2-1042.9) (Figure 2A), highest for Lynch syndrome in Cherokee (4838.7; 95% CI, 1744.2-13 916.72) vs Charleston (320.0; 95% CI, 239.3-443.2) (Figure 2B), and highest for familial hypercholesterolemia in Jasper (2304.1; 95% CI, 1016.3-5426.4) vs Charleston (512; 95% CI, 408.4-664.9) (Figure 2C). Interactive county-level data visualizations illustrating screening coverage per 100 000 and positivity prevalence per 100 000 are available on the MUSC dashboard.^10^

### In Our DNA SC Enrollment and Screening Coverage by Rurality and Social Disadvantage

In South Carolina, 18 of 46 counties are classified as RUC code 2 (metropolitan areas with populations of 250 000-1 000 000), and there are no counties assigned RUC codes 5 or 7 (eTables 4 and 5 in Supplement 1). The distribution of the 46 South Carolina counties by overall SVI is as follows: 12 counties in quartile 1 (least disadvantaged), 11 in quartile 2, 11 in quartile 3, and 12 in quartile 4 (most disadvantaged) (eTables 4 and 6 in Supplement 1). Findings shown in eTable 7 in Supplement 1 illustrate the likelihood of withdrawal after initial enrollment; no significant differences in withdrawal were observed by rurality or social disadvantage.

The absolute number of participants enrolled and screened and the screening rate per 100 000 population by rurality and social disadvantage are presented in Table 1, and population sizes by RUC codes and SVI quartile are available in eTables 5 and 6 in Supplement 1, respectively. The largest number of participants who enrolled were from RUC code 2 (59 545) counties (metropolitan areas with populations of 250 000-1 000 000) and SVI quartile 1 (47 474) counties (least disadvantaged). Screening rates exceeded 1000 per 100 000 population in RUC code 1 through 3 counties and were significantly higher than RUC code 6 (791.4; 95% CI, 757.1-827.2), RUC code 8 (861.4; 95% CI, 801.1-926.2), and RUC code 9 (839.6; 95% CI, 683.6-1030.8) counties. Screening coverage by overall SVI score was significantly higher in quartile 1 (1422.1; 95% CI, 1406.3-1438.1) and quartile 2 (1188.4; 95% CI, 1167.3-1209.9) compared with quartile 3 (888.4; 95% CI, 866.5-910.9) and quartile 4 (839.6; 95% CI, 952.3-1025.4).

**Table 1.** Screening Rates by Rurality and Social Disadvantage, In Our DNA SC Program

Measure	No. of counties	Total No. of participants enrolled	Participants screened	
			No. (%)^a^	Per 100 000 (95% CI)^b^
**Rurality, RUC code^c^**				
1 (Most urban)	3	3974	2263 (56.9)	1342.8 (1288.9-1398.8)
2	18	59 545	37 606 (63.2)	1357.7 (1344.2-1371.4)
3	5	9442	5823 (61.7)	1400.7 (1365.4-1436.8)
4	4	4130	2450 (59.3)	1036.2 (996.2-1077.8)
5	0	NA	NA	NA
6	9	3360	1942 (57.8)	791.4 (757.1-827.2)
7	0	NA	NA	NA
8	6	1242	723 (58.2)	861.4 (801.1-926.2)
9 (Most rural)	1	185	90 (48.6)	839.6 (683.6-1030.8)
**Social Vulnerability Index, quartiles^d^**				
Overall				
1 (Least disadvantage)	12	47 474	30 196 (63.6)	1422.1 (1406.3-1438.1)
2	11	19 115	11 830 (61.9)	1188.4 (1167.3-1209.9)
3	11	10 080	6088 (60.4)	888.4 (866.5-910.9)
4 (Most disadvantage)	12	5209	2783 (53.4)	988.1 (952.3-1025.4)
Housing characteristics				
1 (Least disadvantage)	12	46 846	29 752 (63.5)	1469.8 (1453.3-1486.4)
2	11	18 145	11 191 (61.7)	995.3 (977.1-1013.8)
3	11	11 980	7325 (61.1)	1096.6 (1071.9-(1121.8)
4 (Most disadvantage)	12	4907	2629 (53.6)	977.2 (940.7-1015.1)
Housing and transportation				
1 (Least disadvantage)	12	34 538	21 954 (63.6)	1517.1 (1497.3-1537.1)
2	11	25 296	15 486 (61.2)	1142.0 (1124.3-1160.0)
3	11	18 984	11 632 (61.3)	1195.9 (1174.5-1217.7)
4 (Most disadvantage)	12	3060	1825 (59.6)	589.0 (562.7-616.6)
Minority status				
1 (Least disadvantage)	12	24 752	15 943 (64.4)	860.5 (847.3-873.9)
2	11	38 369	24 236 (63.2)	1895.9 (1872.4-1919.6)
3	11	7611	4362 (57.3)	1318.9 (1280.5-1358.3)
4 (Most disadvantage)	12	11 146	6356 (57.0)	1018.7 (994.1-1043.9)
Socioeconomic status				
1 (Least disadvantage)	11	38 160	23 918 (62.7)	1356.7 (1339.7-1373.9)
2	11	24 901	16 048 (64.4)	1312.4 (1292.3-1332.7)
3	12	13 775	8232 (59.8)	1005.3 (983.9-1027.1)
4 (Most disadvantage)	12	5042	2699 (53.5)	960.4 (925.0-997.2)

Abbreviations: NA, not applicable; RUC, rural-urban continuum.

^a^ Proportion of screened participants of the total enrolled.

^b^ Rate was calculated per 100 000 adult population aged 18 years or older. Wilson 95% CIs for screening rates per 100 000.

^c^ The RUC codes are based on metropolitan population size, degree of urbanization, and proximity to metropolitan areas. They distinguish metropolitan counties by the size of their metropolitan area and nonmetropolitan counties by their level of urbanization and proximity to a metropolitan area.

^d^ Social Vulnerability Index: quartile 1, 0.00 to 0.25; quartile 2, 0.51 to 0.75; quartile 3, 0.26 to 0.50; quartile 4, 0.76 to 1.00.

### In Our DNA SC Test Positivity by Rurality and Social Disadvantage

Table 2 presents the number of valid and positive test results and test positivity prevalence per 100 000 valid results by RUC codes and SVI quartiles. Across all RUC codes, test positivity in South Carolina exceeded 250 per 100 000 valid results for HBOC and Lynch syndrome and 400 per 100 000 valid results for familial hypercholesterolemia. Positivity prevalence for all 3 tests was nearly similar across rurality, except for HBOC. Compared with RUC code 1 (268.5; 95% CI, 123.1-584.5), HBOC test positivity was significantly higher in RUC code 2 (801.3; 95% CI, 715.3-897.4). When examined by overall SVI quartiles, prevalence of HBOC, Lynch syndrome, and familial hypercholesterolemia remained relatively consistent across quartiles, with no statistically significant differences.

**Table 2.** Test Positivity by Rurality and Social Disadvantage, In Our DNA SC Program

Measure	Positive results							
	No. of counties	Total valid results	HBOC syndrome		Lynch syndrome		Familial hypercholesterolemia	
			No. (%)^a^	Per 100 000 (95% CI)^b^	No. (%)^a^	Per 100 000 (95% CI)^b^	No. (%)^a^	Per 100 000 (95% CI)^b^
**Rurality, RUC code^c^**								
1 (Most urban)	3	2235	6 (0.3)	268.5 (123.1-584.5)	6 (0.3)	268.5 (123.1-584.5)	11 (0.5)	492.2 (275.0-879.2)
2	18	36 942	296 (0.8)	801.3 (715.3-897.4)	131 (0.4)	354.6 (298.9-420.6)	193 (0.5)	522.4 (453.9-601.3)
3	5	5722	38 (0.7)	664.1 (484.2-910.2)	20 (0.3)	349.5 (226.4-539.3)	33	576.7 (411.0-808.8)
4	4	2417	11 (0.5)	455.1 (254.3-813.1)	8 (0.3)	331.0 (167.8-651.8)	10 (0.4)	413.7 (224.9-760.0)
5	0	NA	NA	NA	NA	NA	NA	NA
6	9	1913	7 (0.4)	365.9 (177.4-753.4)	12 (0.6)	627.3 (359.2-1093.3)	8 (0.4)	418.2 (212.1-823.1)
7	0	NA	NA	NA	NA	NA	NA	NA
8	6	714	4 (0.6)	560.2 (218.1-1431.5)	2 (0.3)	NR	3 (0.4)	420.2 (143.0-1228.0)
9 (Most rural)	1	89	0	NR	0	NR	1	NR
**Social Vulnerability Index, quartiles** ^d^								
Overall								
1 (Least disadvantage)	12	29 690	222 (0.7)	747.7 (655.9-852.3)	95 (0.3)	320.0 (261.8-391.0)	159 (0.5)	535.5 (458.7-625.2)
2	11	11 626	83 (0.7)	713.9 (576.3-884.1	52 (0.4)	447.3 (341.3-586.0)	53 (0.5)	455.9 (348.7-595.8)
3	11	5971	44 (0.7)	736.9 (549.4-987.7)	21 (0.4)	351.7 (230.2-537.1)	36 (0.6)	602.9 (435.8-833.5)
4 (Most disadvantage)	12	2745	13 (0.5)	473.6 (277.0-808.6)	11 (0.4)	400.7 (223.9-716.2)	11 (0.4)	400.7 (223.9-716.2)
Housing characteristics								
1 (Least disadvantage)	12	29 253	219 (0.7)	748.6 (656.1-854.1)	93 (0.3)	317.9 (259.6-389.3)	157 (0.5)	536.7 (459.2-627.2)
2	11	10 987	81 (0.7)	737.2 (593.6-915.3)	43 (0.4)	391.4 (290.7-526.7)	58 (0.5)	527.9 (408.6-681.8)
3	11	7200	47 (0.7)	652.8 (491.3-866.9)	35 (0.5)	486.1 (349.7-675.3)	33 (0.5)	458.3 (326.6-643.0)
4 (Most disadvantage)	12	2592	15 (0.6)	578.7 (351.0-952.7)	8 (0.3)	308.6 (156.5-607.9)	11 (0.4)	424.4 (237.1-758.4)
Housing and transportation								
1 (Least disadvantage)	12	21 561	183 (0.8)	848.8 (734.8-980.3)	70 (0.3)	324.7 (257.1-409.9)	112 (0.5)	519.5 (431.9-624.6)
2	11	15 242	91 (0.6)	597.0 (486.6-732.4)	55 (0.4)	360.8 (277.4-469.4)	83 (0.5)	544.5 (439.5-674.5)
3	11	11 423	82 (0.7)	717.8 (578.7-890.1)	47 (0.4)	411.5 (309.6-546.7)	59 (0.5)	516.5 (400.7-665.6)
4 (Most disadvantage)	12	1806	6 (0.3)	332.2 (152.3-723.0)	7 (0.4)	387.6 (187.9-797.9)	5 (0.3)	276.9 (118.3-646.5)
Minority status								
1 (Least disadvantage)	12	15 698	105 (0.7)	668.9 (552.9-809.0)	50 (0.3)	318.5 (241.7-419.6)	72 (0.5)	458.7 (364.4-577.2)
2	11	23 800	187 (0.8)	785.7 (681.2-906.1)	92 (0.4)	386.6 (315.3-473.8)	130 (0.5)	546.2 (460.2-648.2)
3	11	4289	27 (0.6)	629.5 (433.0-914.4)	11 (0.3)	256.5 (143.3-458.7)	29 (0.7)	676.1 (471.2-969.4)
4 (Most disadvantage)	12	6245	43 (0.7)	688.6 (511.6-926.1)	26 (0.4)	416.3 (284.3-609.4)	28 (0.4)	448.4 (310.4-647.3)
Socioeconomic status								
1 (Least disadvantage)	11	23 522	163 (0.7)	693.0 (594.7-807.3)	84 (0.4)	357.1 (288.6-441.9)	120 (0.5)	510.2 (426.8-609.6)
2	11	15 772	125 (0.8)	792.5 (665.6-943.4	54 (0.3)	342.4 (262.5-446.4)	81 (0.5)	513.6 (413.4-637.8)
3	12	8080	62 (0.8)	767.3 (599.1-982.4)	30 (0.4)	371.3 (260.2-529.5)	43 (0.5)	532.2 (395.3-716.0)
4 (Most disadvantage)	12	2658	12 (0.5)	451.5 (258.4-787.5)	11 (0.4)	413.8 (231.2-739.6)	15 (0.6)	564.3 (342.3-929.1)

Abbreviations: HBOC, hereditary breast and ovarian cancer syndrome; NA, not applicable; NR, not reported; RUC, rural-urban continuum.

^a^ Proportion of positive results of the total valid test results.

^b^ Test positivity prevalence was calculated per 100 000 valid test results and required a minimum of 3 positive results. Wilson 95% CIs for screening rates per 100 000.

^c^ The RUC codes are based on metropolitan population size, degree of urbanization, and proximity to metropolitan areas. They distinguish metropolitan counties by the size of their metropolitan area and nonmetropolitan counties by their level of urbanization and proximity to a metropolitan area.

^d^ Social Vulnerability Index: quartile 1, 0.00 to 0.25; quartile 2, 0.51 to 0.75; quartile 3, 0.26 to 0.50; quartile 4, 0.76 to 1.00.

## Discussion

This cross-sectional study shows the statewide reach and broad geographic penetration of the In Our DNA SC program, underscoring the feasibility of implementing a large-scale PWGS across diverse rural-urban and social disadvantage contexts. Critically, the deliberate application of the RE-AIM implementation science framework was central to reaching the more than 80 000 enrollments, providing a structured approach to evaluating reach, effectiveness, adoption, implementation, and maintenance in clinical and community settings. Coupled with this framework, an integrated informatics infrastructure enabled continuous monitoring of program performance and assessment of equitable reach across rurality and social disadvantage levels, thereby informing data-driven adaptations of PWGS.

The enrollment of more than 82 400 participants so far from all 46 South Carolina counties reflects substantial reach and broad representativeness of counties by rurality and social disadvantage. The program’s flexible delivery model, including clinic-based, community-based, and at-home enrollment options, was instrumental in achieving statewide coverage. This multimodal delivery approach may have reduced structural barriers associated with transportation, clinical access, competing life demands, and other factors that disproportionately affect rural and high-SVI communities. From an implementation perspective, site-level adaptations and flexible sample collection strategies enabled consistent delivery while maintaining program fidelity.^7^ The implementation metrics provided critical feedback for continuous quality improvement and illustrated how implementation science frameworks could move beyond participation counts to capture community and clinical delivery challenges that influence effectiveness and sustainability. Beyond clinical benefits, the initiative is generating a rich informatics resource for monitoring program reach (enrollment) and effectiveness (screening completion) through the novel linkage of genomic results with demographic and geographic data, illuminating preliminary insights into test positivity patterns.

Screening coverage rates for the In Our DNA SC program reached or exceeded 800 per 100 000 population across both RUC and SVI strata, indicating strong engagement across diverse communities. Positivity prevalence of HBOC was significantly higher in RUC code 2 compared with RUC code 1 counties, while prevalence for all 3 tests was nearly similar across SVIs. The increased detection of pathogenic variants in RUC code 2 counties suggests that genomic screening may be uncovering a disproportionate burden of previously undiagnosed hereditary risk. These data may highlight true differences in underlying genetic risk or may instead reflect differences in genomic screening access or behaviors, and they merit further investigation. However, equitable access to screening is only 1 component of an effective PWGS program. South Carolina faces substantial structural challenges, including poor overall health rankings, medically underserved regions, and a sizable uninsured population, that may limit individuals’ ability to act on genetic findings.^11^ Ensuring that all participants can obtain appropriate follow-up care will be essential for achieving equitable outcomes as the program continues to expand.

A key strength of this program is the integration of its data into the South Carolina–Cancer Surveillance for Population Health Research and Outreach Tool informatics platform, which enabled monitoring of implementation outcomes, ie, reach and effectiveness, across geographic and social contexts.^10^ By geolinking deidentified participant data with RUC codes and SVI measures, the dashboard operationalizes RE-AIM constructs in a way that is actionable for both researchers and stakeholders. The interactive visualization enhances transparency; facilitates data dissemination; and supports decision-making by health systems, policymakers, and community partners. The ability to visualize enrollment and positivity prevalence per 100 000 population facilitated equitable comparisons across counties and informed targeted outreach and resource allocation. Importantly, the dashboards and findings from this study highlight the need to further improve enrollment and screening in rural counties (RUC codes >4) and counties with high disadvantage (SVI 3 and 4), given that screening coverage exceeded 1000 per 100 000 and was significantly higher compared with urban counties (RUC codes 1-4) and counties with lower disadvantage (SVI 1 and 2). The approach illustrates how informatics infrastructure could serve as a bridge between implementation science and population health practice, enabling adaptive management of large-scale genomic initiatives in US settings. Notably, only 1 prior study from Australia has described the design and implementation of PWGS.^12^ The dual leverage of implementation and informatics in the In Our DNA SC program could offer a promising model for integrating genomics into health systems across the US. Future PWGS efforts and subsequent iterations of the In Our DNA SC dashboard could further advance the informatics infrastructure by incorporating zip code–level or census tract–level data and integrating effectiveness and maintenance metrics, thereby enhancing the program’s ability to more fully capture and assess program equity.

### Limitations

Several limitations should be considered when interpreting these findings. First, although the program achieved statewide reach, participation was voluntary and primarily occurred through outreach within a specific health system, which may have introduced selection bias and limited representativeness of enrolled individuals compared with the underlying county populations, particularly among groups with lower health literacy or limited engagement with health systems. Overall, the proportion of consented participants who provided a sample by July 2025 was 62%, which further underscores the potential for participation bias and the likelihood that those who completed sampling differed systematically from those who did not. Second, coverage and positivity were assessed using aggregated, county-level data, which may have obscured within-county heterogeneity and limited the ability to draw individual-level inferences regarding access, risk, and follow-up care. In addition, individuals with personal or family histories of cancer or cardiovascular disease may have been more likely to participate in screening. The observed test positivity prevalence may have been influenced by indication bias and, therefore, may not reflect true population-level prevalence. Finally, elevated positivity observed in some low-population counties may reflect small denominators and potential founder effects,^13^ underscoring the need for cautious interpretation and further investigation to distinguish true genetic clustering from statistical instability.

## Conclusions

This cross-sectional study of a statewide PWGS program suggests that PWGS could be implemented at scale and achieve broad reach when guided by an implementation science framework and supported by a robust informatics infrastructure. The program reached all 46 counties in South Carolina, showing enrollment and screening coverage across gradients of rurality and social disadvantage while also highlighting areas for improvement. Evaluating clinical and behavioral outcomes remains a critical next step for determining program effectiveness and community and clinical impact. As PWGS efforts expand nationally, integrating RE-AIM–informed evaluation with stakeholder-centered informatics platforms may be essential for translating genomic innovations into sustainable and more equitable population health programs.

## Notes

Supplement 1. **eFigure.** RE-AIM (Reach, Effectiveness, Adoption, Implementation, and Maintenance) Framework Adaptation for In Our DNA SC **eTable 1.** Characteristics of In Our DNA SC Screened Participants **eTable 2.** List of Pathogenic and Likely Pathogenic Variants Identified Among In Our DNA SC Screened Participants **eTable 3.** Demographic Distribution, Cancer Incidence, and Hypercholesterolemia Prevalence in South Carolina Counties **eTable 4.** Urban-Rural Continuum (RUC) Classification, Social Vulnerability, and Population Sizes of South Carolina Counties **eTable 5.** Population Sizes of South Carolina Counties by Urban-Rural Continuum (RUC) Classification **eTable 6.** Population Sizes of South Carolina by County Social Disadvantage **eTable 7.** Logistic Regression Model Evaluating Withdrawal After Initial Consent, In Our DNA SC

Supplement 2.
Data Sharing Statement
