# Supplementary material for: Geographic and Social Equity in Population-Wide Genomic Screening
Source: JAMA Netw Open. 2026 Jul 13;9(7):e2622743. doi: 10.1001/jamanetworkopen.2026.22743 (PMC13366200; doi:10.1001/jamanetworkopen.2026.22743)
Supplement: Supplement 1. — eFigure. RE-AIM (Reach, Effectiveness, Adoption, Implementation, and Maintenance) Framework Adaptation for In Our DNA SC eTable 1. Characteristics of In Our DNA SC Screened Participants eTable 2. List of Pathogenic and Likely Pathogenic Variants Identified Among In Our DNA SC Screened Participants eTable 3. Demographic Distribution, Cancer Incidence, and Hypercholesterolemia Prevalence in South Carolina Counties eTable 4. Urban-Rural Continuum (RUC) Classification, Social Vulnerability, and Population Sizes of South Carolina Counties eTable 5. Population Sizes of South Carolina Counties by Urban-Rural Continuum (RUC) Classification eTable 6. Population Sizes of South Carolina by County Social Disadvantage eTable 7. Logistic Regression Model Evaluating Withdrawal After Initial Consent, In Our DNA SC [file jamanetwopen-e2622743-s001.pdf]

Supplemental Online Content

Sonawane KA, Judge DP, Moore E, Norman S, Allen CG. Geographic and social equity in population-wide genomic screening. *JAMA Netw Open*. 2026;9(7):e2622743. doi:10.1001/jamanetworkopen.2026.22743

eFigure. RE-AIM (Reach, Effectiveness, Adoption, Implementation, and Maintenance) Framework Adaptation for In Our DNA SC

eTable 1. Characteristics of In Our DNA SC Screened Participants

eTable 2. List of Pathogenic and Likely Pathogenic Variants Identified Among In Our DNA SC Screened Participants

eTable 3. Demographic Distribution, Cancer Incidence, and Hypercholesterolemia Prevalence in South Carolina Counties

eTable 4. Urban-Rural Continuum (RUC) Classification, Social Vulnerability, and Population Sizes of South Carolina Counties

eTable 5. Population Sizes of South Carolina Counties by Urban-Rural Continuum (RUC) Classification

eTable 6. Population Sizes of South Carolina by County Social Disadvantage

eTable 7. Logistic Regression Model Evaluating Withdrawal After Initial Consent, In Our DNA SC

This supplemental material has been provided by the authors to give readers additional information about their work.

© 2026 Sonawane KA et al. *JAMA Network Open*.

eFigure. RE-AIM (Reach, Effectiveness, Adoption, Implementation, and Maintenance) Framework Adaptation for *In Our DNA SC*.

The figure outlines the RE-AIM framework used to evaluate the *In Our DNA SC* program, highlighting five key domains: Reach, Effectiveness, Adoption, Implementation, and Maintenance. Each domain includes a definition and specific measures to assess program performance. The structured approach supports a comprehensive evaluation of how well the program engages diverse communities and delivers equitable genomic screening across South Carolina.

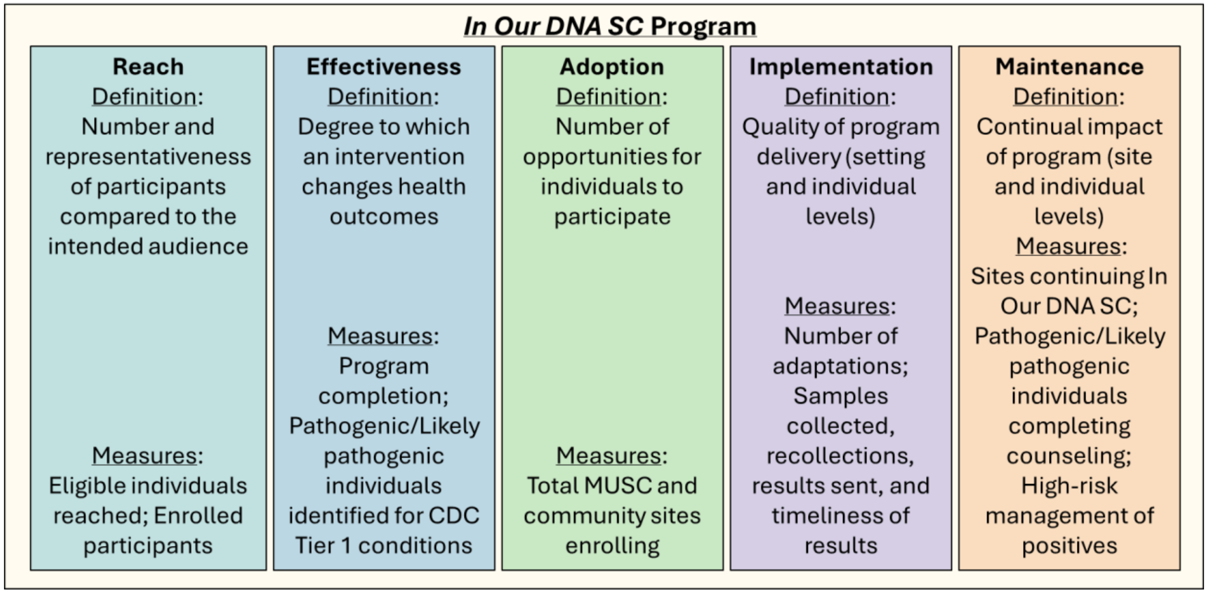

Abbreviations: CDC, Centers for Disease Control and Prevention; MUSC, Medical University of South Carolina.

© 2026 Sonawane KA et al. *JAMA Network Open*.

eTable 1. Characteristics of *In Our DNA SC* Screened Participants

Characteristics	*In Our DNA SC* n=50,897	South Carolina N=4,326,720	Standardized difference
**Age**			0.28
<65 years	36,431 (71.6%)	2,522,478 (58.3)	
≥65 years	14,466 (28.4%)	1,804,242 (20.6)	
**Sex**			-0.42
Male	13,959 (27.4%)	2,075,066 (48.0)	
Female	36,926 (72.6%)	2,251,694 (52.0)	
Unknown	12 (0.02%)		
**Race**			-0.43
Black	4,745 (9.3%)	1,111,967 (25.7)	
White	37,204 (73.1%)	3,214,753 (69.0)	
Other**	8,948 (17.6%)		
**Ethnicity**			-0.24
Hispanic or Latino	1,265 (2.5%)	341,810 (7.9)	
Not Hispanic or Latino	41,339 (81.2%)	3.984,909 (92.0)	
Unknown	8,293 (16.3%)		

*Data from the US Census Bureau, Population Division. Estimates are for the population 18 years from Census 2023 5-year estimate.
**Other races include American Indian/ Alaskan Native, Asian Indian, Chinese, Japanese, Vietnamese, Other Asian, Filipino, Guamanian or Chamorro, Native Hawaiian, Samoan, or more than one race.

© 2026 Sonawane KA et al. *JAMA Network Open*.

eTable 2. List of Pathogenic and Likely Pathogenic Variants Identified Among *In Our DNA SC* Screened Participants

Gene	Variant	Classification	Transcript ID	Protein	n
APOB	c.10579C>T	P	NM_000384.2	p.Arg3527Trp	2
APOB	c.10579C>T	P	NM_000384.3	p.Arg3527Trp	1
APOB	c.10580G>A	P	NM_000384.2	p.Arg3527Gln	38
APOB	c.10580G>A	P	NM_000384.3	p.Arg3527Gln	28
APOB	c.241C>T	P	NM_000527.4	p.Arg3527Gln	1
BRCA1	c.3026C>A	LP	NM_007294.3	p.Ser1009Ter	1
BRCA1	c.1082_1092del	P	NM_007294.3	p.Ser361Ter	2
BRCA1	c.1175_1214del	P	NM_007294.4	p.Leu392GlnfsTer5	1
BRCA1	c.1281_1288del	LP	NM_007294.4	p.Lys428LeufsTer5	1
BRCA1	c.1340_1341insG	P	NM_007294.3	p.His448SerfsTer8	2
BRCA1	c.135-1G>T	P	NM_007294.3		1
BRCA1	c.140G>A	P	NM_007294.3	p.Cys47Tyr	1
BRCA1	c.1677del	P	NM_007294.4	p.Asp560IlefsTer12	1
BRCA1	c.1687C>T	P	NM_007294.4	p.Gln563Ter	1
BRCA1	c.181T>G	P	NM_007294.3	p.Cys61Gly	3
BRCA1	c.181T>G	P	NM_007294.4	p.Cys61Gly	1
BRCA1	c.1823_1826delAGAA	P	NM_007294.3	p.Lys608IlefsTer3	2
BRCA1	c.190T>G	P	NM_007294.4	p.Cys64Gly	1
BRCA1	c.2043dup	P	NM_007294.3	p.Asn682Ter	1
BRCA1	c.2105dup	P	NM_007294.3	p.Leu702PhefsTer10	1
BRCA1	c.213-11T>G	P	NM_007294.3		12
BRCA1	c.213-11T>G	P	NM_007294.4	p.?	2
BRCA1	c.2308_2311delinsCAC	LP	NM_007294.3	p.Ser770HisfsTer22	1
BRCA1	c.2457del	P	NM_007294.3	p.Asp821IlefsTer25	5
BRCA1	c.2457del	P	NM_007294.4	p.Asp821IlefsTer25	2

© 2026 Sonawane KA et al. *JAMA Network Open*.

BRCA1	c.2475del	P	NM_007294.4	p.Asp825GlufsTer21	1
BRCA1	c.2517_2518del	P	NM_007294.4	p.His839GlnfsTer12	1
BRCA1	c.2517_2518delCA	P	NM_007294.3		1
BRCA1	c.2619_2620delinsCTGC	P	NM_007294.4	p.Asn874CysfsTer20	1
BRCA1	c.2646_2648delTGC	P	NM_007294.3	p.Cys882Ter	1
BRCA1	c.2679_2682delGAAA	P	NM_007294.3		1
BRCA1	c.2722G>T	P	NM_007294.3	p.Glu908Ter	1
BRCA1	c.2722G>T	P	NM_007294.4	p.Glu908Ter	1
BRCA1	c.2834_2836delinsC	P	NM_007294.3	p.Ser945ThrfsTer6	1
BRCA1	c.2915del	P	NM_007294.4	p.Gly972AspfsTer28	1
BRCA1	c.2940del	P	NM_007294.3	p.Pro981HisfsTer19	1
BRCA1	c.2940delA	P	NM_007294.3	p.Pro981HisfsTer19	1
BRCA1	c.3005del	P	NM_007294.3	p.Asn1002ThrfsTer22	1
BRCA1	c.303T>G	P	NM_007294.3	p.Tyr101Ter	1
BRCA1	c.3228_3229del	P	NM_007294.3	p.Gly1077AlafsTer8	1
BRCA1	c.3481_3491del	P	NM_007294.3	p.Glu1161PhefsTer3	1
BRCA1	c.3481_3491del	P	NM_007294.4	p.Glu1161PhefsTer3	1
BRCA1	c.3481_3491del11	P	NM_007294.3	p.Glu1161PhefsTer3	2
BRCA1	c.3627dup	P	NM_007294.3	p.Glu1210ArgfsTer9	1
BRCA1	c.3748G>T	P	NM_007294.3	p.Glu1250Ter	4
BRCA1	c.3756_3759del	P	NM_007294.4	p.Ser1253ArgfsTer10	3
BRCA1	c.4057G>T	P	NM_007294.3	p.Glu1353X	1
BRCA1	c.4065_4068del	P	NM_007294.3	p.Asn1355LysfsTer10	2
BRCA1	c.4065_4068del	P	NM_007294.4	p.Asn1355LysfsTer10	2
BRCA1	c.427G>T	P	NM_007294.3	p.Glu143Ter	2
BRCA1	c.4327C>T	P	NM_007294.3	p.Arg1443Ter	4
BRCA1	c.4327C>T	P	NM_007294.4	p.Arg1443Ter	1
BRCA1	c.4357+1G>A	P	NM_007294.4	p.?	1

© 2026 Sonawane KA et al. *JAMA Network Open*.

BRCA1	c.4603G>T	P	NM_007294.3	p.Glu1535Ter	1
BRCA1	c.4612C>T	P	NM_007294.3	p.Gln1538Ter	2
BRCA1	c.4612C>T	P	NM_007294.4	p.Gln1538Ter	2
BRCA1	c.4675+1G>A	LP	NM_007294.3	p.?	1
BRCA1	c.4868C>G	LP	NM_007294.3	p.Ala1623Gly	1
BRCA1	c.4964_4982del	P	NM_007294.4	p.Ser1655TyrfsTer16	1
BRCA1	c.4986+4A>C	LP	NM_007294.3		1
BRCA1	c.5096G>A	P	NM_007294.3	p.Arg1699Gln	9
BRCA1	c.5096G>A	P	NM_007294.4	p.Arg1699Gln	1
BRCA1	c.5123C>A	P	NM_007294.3	p.Ala1708Glu	1
BRCA1	c.5177_5180del	P	NM_007294.4	p.Arg1726LysfsTer3	1
BRCA1	c.5266dup	P	NM_007294.3	p.Gln1756ProfsTer74	4
BRCA1	c.5266dup	P	NM_007294.4	p.Gln1756ProfsTer74	4
BRCA1	c.5266dupC	P	NM_007294.3	p.Gln1756ProfsTer74	5
BRCA1	c.5324T>G	P	NM_007294.3	p.Met1775Arg	1
BRCA1	c.5407-25T>A	LP	NM_007294.4		1
BRCA1	c.5497G>A	P	NM_007294.3	p.Val1833Met	1
BRCA1	c.671-215_901del	LP	NM_007294.3		1
BRCA1	c.68_69del	P	NM_007294.3	p.Glu23ValfsTer17	6
BRCA1	c.68_69del	P	NM_007294.4	p.Glu23ValfsTer17	6
BRCA1	c.68_69delAG	P	NM_007294.3	p.Glu23ValfsTer17	6
BRCA1	c.81-2A>C	LP	NM_007294.3		1
BRCA1	Exon 1-2 of 23 (deletion)	P	NM_007294.4		1
BRCA1	Exon 1-16 of 23 (deletion)	LP	NM_007294.4		1
BRCA1	Exon 1-23 of 23 (deletion)	P	NM_007294.4		1
BRCA1	Exon 12-14 of 23 (deletion)	P	NM_007294		1
BRCA1	Exon 12 of 23 (duplication)	P	NM_007294		2
BRCA1	Exon 12 of 23 (duplication)	LP	NM_007294		1

© 2026 Sonawane KA et al. *JAMA Network Open*.

BRCA1	Exon 12 of 23 (gain)	P	NM_007294.4		1
BRCA1	Exon 15 of 23 (deletion)	P	NM_007294		1
BRCA1	Exon 16 of 23 (deletion)	P	NM_007294.4		1
BRCA1	Exon 17-19 of 23 (duplication)	P	NM_007294		1
BRCA1	Exon 17-19 of 23 (duplication)	LP	NM_007294		1
BRCA1	Exon 20-23 of 23 (deletion)	P	NM_007294		5
BRCA1	Exon 23 of 23 (deletion)	P	NM_007294		1
BRCA2	c.4478_4481del	P	NM_000059.3	p.Glu1493ValfsTer10	1
BRCA2	c.1138del	P	NM_000059.4	p.Ser380ValfsTer19	1
BRCA2	c.1147del	P	NM_000059.3	p.Ile383SerfsTer16	2
BRCA2	c.1147del	P	NM_000059.4	p.Ile383SerfsTer16	1
BRCA2	c.1156G>T	LP	NM_000059.3	p.Glu386Ter	1
BRCA2	c.1265del	P	NM_000059.3	p.Asn422IlefsTer8	1
BRCA2	c.1310_1313del	P	NM_000059.3	p.Lys437IlefsTer22	1
BRCA2	c.1389_1390del	P	NM_000059.3	p.Val464GlyfsTer3	1
BRCA2	c.1499del	P	NM_000059.4	p.Gly500ValfsTer9	1
BRCA2	c.1593dupA	LP	NM_000059.3	p.Glu532ArgfsTer3	1
BRCA2	c.1654delT	P	NM_000059.3	p.Ser552ProfsTer6	1
BRCA2	c.1796_1800del	P	NM_000059.4	p.Ser599Ter	1
BRCA2	c.1813dup	P	NM_000059.3	p.Ile605AsnfsTer11	1
BRCA2	c.1813dupA	P	NM_000059.3	p.Ile605AsnfsTer11	1
BRCA2	c.1832C>A	P	NM_000059.3	p.Ser611Ter	1
BRCA2	c.1929delG	P	NM_000059.3	p.Arg645GlufsTer15	1
BRCA2	c.2330dup	P	NM_000059.4	p.Asp777GlufsTer11	1
BRCA2	c.2368G>T	LP	NM_000059.3	p.Glu790Ter	1
BRCA2	c.2588dup	P	NM_000059.3	p.Asn863LysfsTer18	1
BRCA2	c.2653_2656del	P	NM_000059.4	p.Asp885MetfsTer9	1
BRCA2	c.2808_2811del	P	NM_000059.3	p.Ala938ProfsTer21	2

© 2026 Sonawane KA et al. *JAMA Network Open*.

BRCA2	c.2830A>T	P	NM_000059.3	p.Lys944Ter	3
BRCA2	c.2830A>T	P	NM_000059.4	p.Lys944Ter	2
BRCA2	c.3075_3076delinsTT	P	NM_000059.4	p.Lys1025delinsAsnTer	1
BRCA2	c.3103G>T	P	NM_000059.3	p.Glu1035X	1
BRCA2	c.3103G>T	P	NM_000059.3	p.Glu1035Ter	3
BRCA2	c.3103G>T	P	NM_000059.4	p.Glu1035Ter	2
BRCA2	c.3170_3174del	P	NM_000059.3	p.Lys1057ThrfsTer8	1
BRCA2	c.3170_3174del	P	NM_000059.4	p.Lys1057ThrfsTer8	1
BRCA2	c.3264dup	P	NM_000059.4	p.Gln1089SerfsTer10	1
BRCA2	c.3545_3546del	P	NM_000059.4	p.Phe1182Ter	2
BRCA2	c.3545_3546del	P	NM_000059.4	c.3545_3546del	1
BRCA2	c.3554_3563del	P	NM_000059.3	p.Thr1185IlefsTer9	1
BRCA2	c.3599_3600del	P	NM_000059.3	p.Cys1200Ter	2
BRCA2	c.3599_3600del	P	NM_000059.4	p.Cys1200Ter	1
BRCA2	c.3599_3600delGT	P	NM_000059.3	p.Cys1200Ter	1
BRCA2	c.3785C>G	P	NM_000059.3	p.Ser1262Ter	1
BRCA2	c.3847_3848del	P	NM_000059.3	p.Val1283LysfsTer2	5
BRCA2	c.3847_3848del	P	NM_000059.4	p.Val1283LysfsTer2	5
BRCA2	c.3847_3848delGT	P	NM_000059.3	p.Val1283LysfsTer2	4
BRCA2	c.4211_4215del	P	NM_000059.3	p.Ser1404Ter	1
BRCA2	c.4211_4215del	P	NM_000059.4	p.Ser1404Ter	1
BRCA2	c.4211del	P	NM_000059.3	p.Ser1404Ter	1
BRCA2	c.4284dup	P	NM_000059.4	p.Gln1429SerfsTer9	2
BRCA2	c.4478_4481del	P	NM_000059.3	p.Glu1493ValfsTer10	2
BRCA2	c.4478_4481del	P	NM_000059.4	p.Glu1493ValfsTer10	1
BRCA2	c.4631del	P	NM_000059.3	p.Asn1544ThrfsTer24	1
BRCA2	c.4638del	P	NM_000059.3	p.Phe1546LeufsTer22	1
BRCA2	c.4638delT	P	NM_000059.3		1

© 2026 Sonawane KA et al. *JAMA Network Open*.

BRCA2	c.4876_4877del	P	NM_000059.3	p.Asn1626SerfsTer12	2
BRCA2	c.4876_4877del	P	NM_000059.4	p.Asn1626SerfsTer12	6
BRCA2	c.489_490insG	P	NM_000059.3	p.Leu164ValfsTer19	1
BRCA2	c.4936_4939del	P	NM_000059.3	p.Glu1646GlnfsTer23	2
BRCA2	c.4936_4939del	P	NM_000059.4	p.Glu1646GlnfsTer23	2
BRCA2	c.4936_4939delGAAA	P	NM_000059.3	p.Glu1646GlnfsTer23	3
BRCA2	c.4965C>A	P	NM_000059.3	p.Tyr1655Ter	2
BRCA2	c.4965C>G	P	NM_000059.3	p.Tyr1655Ter	6
BRCA2	c.4965C>G	P	NM_000059.4	p.Tyr1655Ter	1
BRCA2	c.5035del	P	NM_000059.4	p.Thr1679LeufsTer3	1
BRCA2	c.5042_5043del	P	NM_000059.4	p.Val1681GlufsTer7	1
BRCA2	c.5065_5066delinsAAA	P	NM_000059.3	p.Ala1689LysfsTer6	1
BRCA2	c.5073dup	P	NM_000059.4	p.Trp1692MetfsTer3	1
BRCA2	c.5103_5106delACCA	LP	NM_000059.3	p.Pro1702LysfsTer3	1
BRCA2	c.5158dup	P	NM_000059.3	p.Ser1720PhefsTer7	1
BRCA2	c.517-2A>G	P	NM_000059.3		2
BRCA2	c.5213_5216del	P	NM_000059.3	p.Thr1738IlefsTer2	1
BRCA2	c.5350_5351del	P	NM_000059.3	p.Asn1784HisfsTer2	3
BRCA2	c.5351dup	P	NM_000059.3	p.Asn1784LysfsTer3	1
BRCA2	c.5576_5579del	P	NM_000059.3	p.Ile1859LysfsTer3	2
BRCA2	c.5576_5579del	P	NM_000059.4	p.Ile1859LysfsTer3	2
BRCA2	c.5616_5620del	P	NM_000059.4	p.Lys1872AsnfsTer2	1
BRCA2	c.5655C>A	P	NM_000059.4	p.Cys1885Ter	1
BRCA2	c.5682C>G	P	NM_000059.3	p.Tyr1894Ter	1
BRCA2	c.5682C>G	P	NM_000059.4	p.Tyr1894Ter	1
BRCA2	c.5722_5723del	P	NM_000059.4	p.Leu1908ArgfsTer2	1
BRCA2	c.574_575del	P	NM_000059.3	p.Met192ValfsTer13	2
BRCA2	c.5782G>T	P	NM_000059.3	p.Glu1928Ter	1

© 2026 Sonawane KA et al. *JAMA Network Open*.

BRCA2	c.5828delC	P	NM_000059.3	p.Ser1943LeufsTer20	1
BRCA2	c.5857G>T	P	NM_000059.4	p.Glu1953Ter	1
BRCA2	c.5864C>A	P	NM_000059.3	p.Ser1955Ter	1
BRCA2	c.5946del	P	NM_000059.3	p.Ser1982ArgfsTer22	5
BRCA2	c.5946del	P	NM_000059.4	p.Ser1982ArgfsTer22	10
BRCA2	c.5946delT	P	NM_000059.3	p.Ser1982ArgfsTer22	6
BRCA2	c.6007del	LP	NM_000059.3	p.Ile2003Ter	1
BRCA2	c.6025C>T	P	NM_000059.3	p.Gln2009Ter	2
BRCA2	c.6082_6086del	P	NM_000059.3	p.Glu2028LysfsTer19	1
BRCA2	c.6220del	LP	NM_000059.3	p.His2074ThrfsTer7	1
BRCA2	c.6220del	P	NM_000059.4	p.His2074ThrfsTer7	1
BRCA2	c.6275_6276del	P	NM_000059.4	p.Leu2092ProfsTer7	1
BRCA2	c.631+2T>G	P	NM_000059.3		2
BRCA2	c.631+2T>G	P	NM_000059.4	p.?	1
BRCA2	c.632-1G>C	LP	NM_000059.4		1
BRCA2	c.6420_6421insA	P	NM_000059.4	p.Gly2141ArgfsTer6	1
BRCA2	c.6444del	P	NM_000059.4	p.Ile2149LeufsTer19	1
BRCA2	c.6449_6450dup	P	NM_000059.4	p.Val2151LysfsTer18	1
BRCA2	c.6486_6489del	P	NM_000059.3	p.Lys2162AsnfsTer5	1
BRCA2	c.658_659del	P	NM_000059.3	p.Val220IlefsTer4	2
BRCA2	c.658_659del	P	NM_000059.4	p.Val220IlefsTer4	1
BRCA2	c.658_659delGT	P	NM_000059.3	p.Val220IlefsTer4	1
BRCA2	c.6580dupA	P	NM_000059.3	p.Ile2194AsnfsTer3	1
BRCA2	c.6644_6647del	P	NM_000059.4	p.Tyr2215SerfsTer13	1
BRCA2	c.6644_6647delACTC	P	NM_000059.3	p.Tyr2215SerfsTer13	1
BRCA2	c.67+1G>T	P	NM_000059.4		1
BRCA2	c.681+1G>A	LP	NM_000059.3		1
BRCA2	c.6952C>T	P	NM_000059.4	p.Arg2318Ter	1

© 2026 Sonawane KA et al. *JAMA Network Open*.

BRCA2	c.7007+5G>A	LP	NM_000059.4		1
BRCA2	c.7007G>A	P	NM_000059.3	p.Arg2336His	1
BRCA2	c.7007G>A	P	NM_000059.4	p.Arg2336His	1
BRCA2	c.7008-2A>G	P	NM_000059.3		2
BRCA2	c.7069_7070del	P	NM_000059.4	p.Leu2357ValfsTer2	1
BRCA2	c.7069_7070delCT	P	NM_000059.3	p.Leu2357ValfsTer2	1
BRCA2	c.7258G>T	P	NM_000059.3	p.Glu2420Ter	1
BRCA2	c.7480C>T	P	NM_000059.3	p.Arg2494Ter	2
BRCA2	c.755_758del	P	NM_000059.3	p.Asp252ValfsTer24	2
BRCA2	c.7558C>T	P	NM_000059.3	p.Arg2520Ter	3
BRCA2	c.7558C>T	P	NM_000059.4	p.Arg2520Ter	2
BRCA2	c.7618-1G>A	P	NM_000059.3		3
BRCA2	c.7762_7764delinsTT	P	NM_000059.4	p.Ile2588PhefsTer60	2
BRCA2	c.778_779del	P	NM_000059.3	p.Glu260SerfsTer15	1
BRCA2	c.778_779del	P	NM_000059.4	p.Glu260SerfsTer15	1
BRCA2	c.7826G>T	LP	NM_000059.3	p.Gly2609Val	1
BRCA2	c.7826G>T	LP	NM_000059.4	p.Gly2609Val	3
BRCA2	c.7879A>T	P	NM_000059.3	p.Ile2627Phe	1
BRCA2	c.7964A>G	LP	NM_000059.3	p.Gln2655Arg	1
BRCA2	c.7964A>G	LP	NM_000059.4	p.Gln2655Arg	1
BRCA2	c.7977-1G>C	P	NM_000059.4		1
BRCA2	c.8009C>T	LP	NM_000059.4	p.Ser2670Leu	1
BRCA2	c.8167G>C	P	NM_000059.3	p.Asp2723His	1
BRCA2	c.8167G>C	P	NM_000059.4	p.Asp2723His	1
BRCA2	c.8169T>A	LP	NM_000059.4	p.Asp2723Glu	1
BRCA2	c.8297del	P	NM_000059.3	p.Thr2766AsnfsTer11	1
BRCA2	c.8350C>T	LP	NM_000059.4	p.Arg2784Trp	1
BRCA2	c.8363G>A	P	NM_000059.4	p.Trp2788Ter	1

© 2026 Sonawane KA et al. *JAMA Network Open*.

BRCA2	c.8537_8538del	P	NM_000059.4	p.Glu2846GlyfsTer22	1
BRCA2	c.8575del	P	NM_000059.4	p.Gln2859LysfsTer4	1
BRCA2	c.8585dup	P	NM_000059.4	p.Glu2863ArgfsTer6	1
BRCA2	c.8754+4A>G	P	NM_000059.4	p.?	1
BRCA2	c.8904delC	P	NM_000059.3	p.Val2969CysfsTer7	1
BRCA2	c.8953+1G>T	P	NM_000059.3		1
BRCA2	c.8954-5A>G	LP	NM_000059.4	p.?	1
BRCA2	c.9026_9030del	P	NM_000059.3	p.Tyr3009SerfsTer7	2
BRCA2	c.9218A>G	LP	NM_000059.3	p.Asp3073Gly	4
BRCA2	c.9253del	P	NM_000059.4	p.Thr3085GlnfsTer19	1
BRCA2	c.9275_9278delATTT	P	NM_000059.3	p.Tyr3092CysfsTer11	1
BRCA2	c.9285C>G	P	NM_000059.3	p.Asp3095Glu	3
BRCA2	c.9294C>G	P	NM_000059.4	p.Tyr3098Ter	1
BRCA2	c.9382C>T	P	NM_000059.4	p.Arg3128Ter	1
BRCA2	c.9403del	P	NM_000059.4	p.Leu3135PhefsTer28	1
BRCA2	c.9435_9436del	P	NM_000059.3	p.Ser3147CysfsTer2	2
BRCA2	c.9502-1G>A	LP	NM_000059.4		1
BRCA2	c.956dupA	P	NM_000059.3		1
BRCA2	c.9648+1G>C	LP	NM_000059.3	p.?	1
BRCA2	c.9648+1G>C	LP	NM_000059.4		1
BRCA2	c.9699_9702del	LP	NM_000059.3	p.Cys3233TrpfsTer15	1
BRCA2	c.9759T>A	LP	NM_000059.3	p.Cys3253X	1
BRCA2	Exon 1-2 of 27 (deletion)	P	NM_000059		5
BRCA2	Exon 1-2 of 27 (deletion)	P	NM_000059.4		1
BRCA2	Exon 1-27 of 27 (deletion)	P	NM_000059.4		1
BRCA2	Exon 14-16 of 27 (deletion)	P	NM_000059.4		1
BRCA2	Exon 12-13 of 27 (duplication)	LP	NM_000059		1
EPCAM	Exon 8-9 of 9 (deletion)	P	NM_002354.3		1

© 2026 Sonawane KA et al. *JAMA Network Open*.

LDLR	c.1003G>A	LP	NM_000527.4	p.Gly335Ser	3
LDLR	c.1003G>A	LP	NM_000527.5	p.Gly335Ser	2
LDLR	c.1027G>A	LP	NM_000527.4	p.Gly343Ser	5
LDLR	c.1027G>A	P	NM_000527.5	p.Gly343Ser	1
LDLR	c.1048C>T	P	NM_000527.5	p.Arg350Ter	1
LDLR	c.1056_1060+3del	LP	NM_000527.5		1
LDLR	c.1061A>T	LP	NM_000527.4	p.Asp354Val	1
LDLR	c.1103G>A	P	NM_000527.4	p.Cys368Tyr	1
LDLR	c.1118_1121dup	P	NM_000527.4	p.Tyr375TrpfsTer7	1
LDLR	c.1118_1121dup	P	NM_000527.5	p.Tyr375TrpfsTer7	1
LDLR	c.1135T>C	P	NM_000527.4	p.Cys379Arg	1
LDLR	c.1187-10G>A	P	NM_000527.4		1
LDLR	c.1187-10G>A	P	NM_000527.4	p.?	1
LDLR	c.1201C>G	LP	NM_000527.4	p.Leu401Val	2
LDLR	c.1201C>G	LP	NM_000527.5	p.Leu401Val	1
LDLR	c.1217G>A	LP	NM_000527.5	p.Arg406Gln	1
LDLR	c.1238C>T	LP	NM_000527.5	p.Thr413Met	1
LDLR	c.1252G>A	LP	NM_000527.4	p.Glu418Lys	1
LDLR	c.1307T>C	LP	NM_000527.4	p.Val436Ala	2
LDLR	c.131G>A	P	NM_000527.4	p.Trp44Ter	1
LDLR	c.1329G>C	P	NM_000527.5	p.Trp443Cys	2
LDLR	c.1359-5C>G	LP	NM_000527.5		1
LDLR	c.1414G>T	LP	NM_000527.4	p.Asp472Tyr	2
LDLR	c.1414G>T	LP	NM_000527.5	p.Asp472Tyr	1
LDLR	c.1432G>A	LP	NM_000527.4	p.Gly478Arg	1
LDLR	c.1444G>A	P	NM_000527.4	p.Asp482Asn	3
LDLR	c.1444G>A	P	NM_000527.5	p.Asp482Asn	1
LDLR	c.1567G>A	LP	NM_000527.4	p.Val523Met	2

© 2026 Sonawane KA et al. *JAMA Network Open*.

LDLR	c.1576C>T	LP	NM_000527.4	p.Pro526Ser	1
LDLR	c.1576C>T	LP	NM_000527.5	p.Pro526Ser	1
LDLR	c.1690A>C	P	NM_000527.4	p.Asn564His	1
LDLR	c.1690A>G	LP	NM_000527.4	p.Asn564Asp	1
LDLR	c.1691A>G	LP	NM_000527.4	p.Asn564Ser	1
LDLR	c.1721G>A	LP	NM_000527.4	p.Arg574His	2
LDLR	c.1721G>A	LP	NM_000527.5	p.Arg574His	3
LDLR	c.1727A>G	LP	NM_000527.4	p.Tyr576Cys	1
LDLR	c.1730G>A	P	NM_000527.4	p.Trp577X	1
LDLR	c.1739C>T	LP	NM_000527.5	p.Ser580Phe	1
LDLR	c.1775G>A	LP	NM_000527.4	p.Gly592Glu	4
LDLR	c.1775G>A	P	NM_000527.5	p.Gly592Glu	5
LDLR	c.1783C>T	P	NM_000527.4	p.Arg595Trp	2
LDLR	c.1783C>T	P	NM_000527.5	p.Arg595Trp	1
LDLR	c.1845+1G>C	P	NM_000527.4		1
LDLR	c.1880C>A	LP	NM_000527.4	p.Ala627Asp	1
LDLR	c.1897C>T	P	NM_000527.4	p.Arg633Cys	12
LDLR	c.1897C>T	P	NM_000527.5	p.Arg633Cys	5
LDLR	c.1898G>A	LP	NM_000527.4	p.Arg633His	1
LDLR	c.190+4A>T	LP	NM_000527.4		3
LDLR	c.1911del	P	NM_000527.4	p.Asp638MetfsTer27	1
LDLR	c.2043C>A	P	NM_000527.4	p.Cys681Ter	2
LDLR	c.2054C>T	P	NM_000527.4	p.Pro685Leu	1
LDLR	c.2054C>T	P	NM_000527.5	p.Pro685Leu	1
LDLR	c.2061dup	P	NM_000527.4	p.Asn688GlnfsTer29	1
LDLR	c.2092del	LP	NM_000527.4	p.Cys698AlafsTer11	1
LDLR	c.2098G>A	LP	NM_000527.4	p.Asp700Asn	6
LDLR	c.2098G>A	LP	NM_000527.5	p.Asp700Asn	4

© 2026 Sonawane KA et al. *JAMA Network Open*.

LDLR	c.2113G>C	LP	NM_000527.5	p.Ala705Pro	2
LDLR	c.214delG	P	NM_000527.4	p.Asp72ThrfsTer134	1
LDLR	c.241C>T	LP	NM_000527.4	p.Arg81Cys	4
LDLR	c.2546C>A	LP	NM_000527.4	p.Ser849Ter	1
LDLR	c.259T>G	LP	NM_000527.4	p.Trp87Gly	1
LDLR	c.301G>A	P	NM_000527.5	p.Glu101Lys	1
LDLR	c.314-2A>C	P	NM_000527.4		1
LDLR	c.337G>A	LP	NM_000527.4	p.Glu113Lys	7
LDLR	c.337G>A	LP	NM_000527.5	p.Glu113Lys	1
LDLR	c.420G>C	P	NM_000527.4	p.Glu140Asp	1
LDLR	c.423del	LP	NM_000527.5	p.Ser142ProfsTer64	1
LDLR	c.494G>A	LP	NM_000527.4	p.Trp165Ter	1
LDLR	c.502G>A	LP	NM_000527.5	p.Asp168Asn	2
LDLR	c.502G>C	P	NM_000527.5	p.Asp168His	1
LDLR	c.513del	P	NM_000527.5	p.Asp172ThrfsTer34	1
LDLR	c.551G>A	LP	NM_000527.4	p.Cys184Tyr	1
LDLR	c.551G>A	P	NM_000527.5	p.Cys184Tyr	1
LDLR	c.589T>C	P	NM_000527.5	p.Cys197Arg	1
LDLR	c.590G>A	P	NM_000527.4	p.Cys197Tyr	1
LDLR	c.632A>T	LP	NM_000527.4	p.His211Leu	1
LDLR	c.632A>T	LP	NM_000527.5	p.His211Leu	1
LDLR	c.660_661delinsAC	LP	NM_000527.5	p.Asp221His	1
LDLR	c.661G>A	P	NM_000527.4	p.Asp221Asn	1
LDLR	c.662A>G	P	NM_000527.4	p.Asp221Gly	1
LDLR	c.663_681dup	LP	NM_000527.4	p.Glu228LeufsTer6	1
LDLR	c.666C>A	P	NM_000527.4	p.Cys222Ter	1
LDLR	c.680_681delAC	P	NM_000527.4	p.Asp227GlyfsTer12	1
LDLR	c.681C>G	P	NM_000527.5	p.Asp227Glu	1

© 2026 Sonawane KA et al. *JAMA Network Open*.

LDLR	c.682G>C	P	NM_000527.5	p.Glu228Gln	1
LDLR	c.682G>T	P	NM_000527.4	p.Glu228Ter	3
LDLR	c.6del	P	NM_000527.4	p.Trp4GlyfsTer202	3
LDLR	c.6del	P	NM_000527.5	p.Trp4GlyfsTer202	3
LDLR	c.718G>A	P	NM_000527.4	p.Glu240Lys	7
LDLR	c.718G>A	P	NM_000527.5	p.Glu240Lys	5
LDLR	c.761A>C	LP	NM_000527.5	p.Gln254Pro	1
LDLR	c.782G>T	LP	NM_000527.5	p.Cys261Phe	1
LDLR	c.798T>A	P	NM_000527.4	p.Asp266Glu	2
LDLR	c.798T>A	P	NM_000527.5	p.Asp266Glu	2
LDLR	c.818-1G>A	P	NM_000527.5		2
LDLR	c.818-1G>T	P	NM_000527.4		1
LDLR	c.820del	P	NM_000527.4	p.Ser274AlafsTer73	1
LDLR	c.858C>A	LP	NM_000527.4	p.Ser286Arg	4
LDLR	c.862G>A	LP	NM_000527.4	p.Glu288Lys	7
LDLR	c.862G>A	P	NM_000527.5	p.Glu288Lys	3
LDLR	c.910G>A	LP	NM_000527.4	p.Asp304Asn	1
LDLR	c.910G>A	P	NM_000527.5	p.Asp304Asn	2
LDLR	c.933del	P	NM_000527.5	p.Glu312SerfsTer58	1
LDLR	c.941-2A>C	LP	NM_000527.5		1
LDLR	c.97C>T	P	NM_000527.4	p.Gln33X	1
LDLR	Exon 1 of 18 (deletion)	P	NM_000527		3
LDLR	Exon 1 of 18 (deletion)	LP	NM_000527.5		2
LDLR	Exon 16 of 18 (deletion)	P	NM_000527.5		1
LDLR	Exon 17-18 of 18 (deletion)	LP	NM_000527		3
LDLR	Exons 17-18 of 18 (deletion)	P	NM_000527.5		3
LDLR	Exon 3-14 of 18 (deletion)	LP	NM_000527		2
LDLR	Exon 8-10 of 18 (deletion)	LP	NM_000527.5		1

© 2026 Sonawane KA et al. *JAMA Network Open*.

LDLRAP1	c.118G>T	LP	NM_015627.2	p.Glu40Ter	1
LDLRAP1	c.135del	LP	NM_015627.2	p.Met46CysfsTer10	1
LDLRAP1	c.143delT	LP	NM_015627.2	p.Phe48SerfsTer8	1
LDLRAP1	c.400C>T	LP	NM_015627.2	p.Gln134Ter	1
LDLRAP1	c.402del	LP	NM_015627.2	p.Ser135AlafsTer27	1
LDLRAP1	c.603dupC	P	NM_015627.2	p.Ser202LeufsTer19	1
LDLRAP1	c.604delinsCC	P	NM_015627.3	p.Ser202ProfsTer19	2
LDLRAP1	c.71_87del	LP	NM_015627.3	p.Gly24GlufsTer4	1
LDLRAP1	c.71del	P	NM_015627.2	p.Gly24Alafs*32	3
LDLRAP1	c.71del	P	NM_015627.2	p.Gly24AlafsTer32	7
LDLRAP1	c.71del	P	NM_015627.3	p.Gly24AlafsTer32	4
LDLRAP1	c.71dup	P	NM_015627.3	p.Gly25ArgfsTer9	2
MLH1	c.117-2A>G	P	NM_000249.3		3
MLH1	c.1667G>C	P	NM_000249.3	p.Ser556Thr	1
MLH1	c.1732-1G>A	P	NM_000249.3		1
MLH1	c.1989+2T>C	LP	NM_000249.3		1
MLH1	c.1A>G	P	NM_000249.4	p.Met1?	1
MLH1	c.2092_2093del	P	NM_000249.4	p.Ser698ArgfsTer5	1
MLH1	c.292G>A	LP	NM_000249.3	p.Gly98Ser	1
MLH1	c.298C>T	P	NM_000249.4	p.Arg100Ter	1
MLH1	c.790+1G>A	P	NM_000249.4	p.?	1
MLH1	Exon 6 of 19 (deletion)	P	NM_000249		1
MLH1	Exon 6-12 of 19 (duplication)	P	NM_000249		1
MSH2	c.-82G>C	LP	NM_000251.3		2
MSH2	c.-82G>C	LP	NM_000251.3	p.?	1
MSH2	c.1216C>T	P	NM_000251.3	p.Arg406Ter	1
MSH2	c.1276+1G>T	LP	NM_000251.2	p?	1
MSH2	c.1369A>C	LP	NM_000251.3	p.Thr457Pro	1

© 2026 Sonawane KA et al. *JAMA Network Open*.

MSH2	c.1393_1420del	P	NM_000251.3	p.Asn465LeufsTer8	1
MSH2	c.1661+1G>A	P	NM_000251.3	p.?	1
MSH2	c.1760-1G>A	LP	NM_000251.3		1
MSH2	c.1838dup	P	NM_000251.2	p.Asn613LysfsTer31	1
MSH2	c.1862G>T	LP	NM_000251.3	p.Arg621Leu	3
MSH2	c.1906G>C	P	NM_000251.3	p.Ala636Pro	1
MSH2	c.2030C>G	LP	NM_000251.3	p.Thr677Arg	5
MSH2	c.2038C>T	P	NM_000251.2	p.Arg680Ter	2
MSH2	c.2060T>C	P	NM_000251.3	p.Leu687Pro	1
MSH2	c.294T>A	P	NM_000251.2	p.Tyr98Ter	1
MSH2	c.463del	P	NM_000251.3	p.Val155LeufsTer19	1
MSH2	c.484G>A	P	NM_000251.3	p.Gly162Arg	2
MSH2	c.942+3A>G	LP	NM_000251.3		1
MSH2	c.942+3A>T	P	NM_000251.3	p.?	1
MSH2	Exon 1-6 of 16 (deletion)	P	NM_000251		1
MSH2	Exon 16 of 16 (deletion)	P	NM_000251		1
MSH2	Exon 5-16 of 16 (deletion)	LP	NM_000251		1
MSH2	Exon 5-7 of 16 (duplication)	LP	NM_000251		1
MSH6	c.10C>T	P	NM_000179.2	p.Gln4Ter	4
MSH6	c.10C>T	P	NM_000179.3	p.Gln4Ter	4
MSH6	c.1108_1109delTT	LP	NM_000179.2	p.Leu370ArgfsTer4	1
MSH6	c.1134_1135del	LP	NM_000179.3	p.Asp380Ter	1
MSH6	c.1168_1170delinsAA	P	NM_000179.3	p.Asp390AsnfsTer21	1
MSH6	c.1263dup	LP	NM_000179.2	p.Asp422Ter	1
MSH6	c.1392del	LP	NM_000179.2	p.Ile464MetfsTer17	1
MSH6	c.1444C>T	P	NM_000179.2	p.Arg482Ter	1
MSH6	c.1634_1635del	LP	NM_000179.2	p.Lys545ArgfsTer17	1
MSH6	c.1634_1635delAA	P	NM_000179.2		1

© 2026 Sonawane KA et al. *JAMA Network Open*.

MSH6	c.1637_1638del	P	NM_000179.2	p.Glu546GlyfsTer16	3
MSH6	c.1705_1706del	P	NM_000179.2	p.Phe569HisfsTer7	2
MSH6	c.1754del	LP	NM_000179.2	p.Leu585GlnfsTer25	1
MSH6	c.1767T>A	P	NM_000179.3	p.Tyr589Ter	1
MSH6	c.2057G>A	P	NM_000179.2	p.Gly686Asp	3
MSH6	c.2230dup	P	NM_000179.3	p.Glu744GlyfsTer12	1
MSH6	c.2290dup	P	NM_000179.3	p.Thr764AsnfsTer8	1
MSH6	c.2314C>T	LP	NM_000179.2	p.Arg772Trp	1
MSH6	c.2338_2339del	LP	NM_000179.2	p.Ala780ProfsTer4	1
MSH6	c.2731C>T	P	NM_000179.2	p.Arg911Ter	3
MSH6	c.2731C>T	P	NM_000179.3	p.Arg911Ter	3
MSH6	c.3226C>G	LP	NM_000179.2	p.Arg1076Gly	1
MSH6	c.3226C>T	LP	NM_000179.2	p.Arg1076Cys	1
MSH6	c.3226C>T	P	NM_000179.3	p.Arg1076Cys	1
MSH6	c.3227G>A	LP	NM_000179.2	p.Arg1076His	2
MSH6	c.3261del	P	NM_000179.2	p.Phe1088SerfsTer2	1
MSH6	c.3261del	P	NM_000179.3	p.Phe1088SerfsTer2	1
MSH6	c.3261dup	P	NM_000179.2	p.Phe1088LeufsTer5	2
MSH6	c.3261dup	P	NM_000179.3	p.Phe1088LeufsTer5	1
MSH6	c.3261dupC	P	NM_000179.2	p.Phe1088LeufsTer5	4
MSH6	c.3261dupC	P	NM_000179.2	p.Phe1088LeufsTer5	1
MSH6	c.3268_3274del7	P	NM_000179.2	p.Glu1090LysfsTer23	1
MSH6	c.3514dup	P	NM_000179.2	p.Arg1172LysfsTer5	1
MSH6	c.3528_3532del	LP	NM_000179.2	p.Leu1177CysfsTer9	1
MSH6	c.3619_3620delCA	P	NM_000179.2		1
MSH6	c.3699_3702del	P	NM_000179.3	p.Lys1233AsnfsTer6	1
MSH6	c.3732_3735dup	LP	NM_000179.2	p.Ser1246IlefsTer30	1
MSH6	c.3743_3744insT	P	NM_000179.2	p.Tyr1249LeufsTer26	1

© 2026 Sonawane KA et al. *JAMA Network Open*.

MSH6	c.377C>G	LP	NM_000179.2	p.Ser126Ter	1
MSH6	c.3984_3987dup	P	NM_000179.3	p.Leu1330ValfsTer12	1
MSH6	c.4001del	LP	NM_000179.2		1
MSH6	c.423del	LP	NM_000179.2	p.Trp142GlyfsTer7	1
MSH6	c.467C>G	P	NM_000179.2	p.Ser156Ter	1
MSH6	c.644dup	P	NM_000179.2	p.Thr216AsnfsTer3	1
MSH6	c.699_702dup	LP	NM_000179.2	p.Thr235Ter	1
MSH6	c.718C>T	P	NM_000179.2	p.Arg240Ter	3
MSH6	c.718C>T	P	NM_000179.3	p.Arg240Ter	1
MSH6	c.741dup	LP	NM_000179.2	p.Arg248ThrfsTer8	1
MSH6	c.892C>T	P	NM_000179.2	p.Arg298X	1
MSH6	c.892C>T	P	NM_000179.3	p.Arg298Ter	2
MSH6	c.908dup	LP	NM_000179.3	p.Met303IlefsTer9	1
MSH6	Exon 2 of 10 (deletion)	LP	NM_000179		1
MSH6	Exon 5-6 of 10 (deletion)	P	NM_000179		1
MSH6	Exon 6-10 of 10 (deletion)	LP	NM_000179		1
MSH6	Exon 6-10 of 10 (deletion)	LP	NM_000179.3		1
PCSK9	c.1120G>T	P	NM_174936.3	p.Asp374Tyr	1
PMS2	c.1036C>T	LP	NM_000535.6	p.Gln346Ter	1
PMS2	c.1111_1112del	P	NM_000535.7	p.Asn371CysfsTer10	1
PMS2	c.123_131del9	LP	NM_000535.6	p.Leu42_Glu44del	1
PMS2	c.137G>T	LP	NM_000535.6	p.Ser46Ile	12
PMS2	c.137G>T	P	NM_000535.6	p.Ser46Ile	1
PMS2	c.137G>T	P	NM_000535.7	p.Ser46Ile	12
PMS2	c.164-1G>C	LP	NM_000535.6		2
PMS2	c.164-1G>C	LP	NM_000535.6	p.?	1
PMS2	c.164-1G>C	LP	NM_000535.7		1
PMS2	c.1A>G	LP	NM_000535.6	p.Met1?	1

© 2026 Sonawane KA et al. *JAMA Network Open*.

PMS2	c.24-12_107delinsAAAT	P	NM_000535.6	p.?	1
PMS2	c.251-2A>T	P	NM_000535.7		1
PMS2	c.251-2A>T	P	NM_000535.7	p.?	1
PMS2	c.2T>A	LP	NM_000535.7	p.Met1?	2
PMS2	c.325del	LP	NM_000535.6	p.Glu109LysfsTer3	1
PMS2	c.400C>T	P	NM_000535.6	p.Arg134Ter	1
PMS2	c.614A>C	LP	NM_000535.6	p.Gln205Pro	1
PMS2	c.614A>C	LP	NM_000535.7	p.Gln205Pro	3
PMS2	c.697C>T	P	NM_000535.6	p.Gln233Ter	1
PMS2	c.706-1G>T	P	NM_000535.6		1
PMS2	c.736_741delinsTGTGTGTGAAG	P	NM_000535.6	p.Pro246CysfsTer3	9
PMS2	c.736_741delinsTGTGTGTGAAG	P	NM_000535.7	p.Pro246CysfsTer3	4
PMS2	c.943C>T	P	NM_000535.6	p.Arg315Ter	3
PMS2	c.943C>T	P	NM_000535.7	p.Arg315Ter	1
PMS2	Exon 1-5 of 15 (deletion)	P	NM_000535		1
PMS2	Exon 1-8 of 15 (deletion)	LP	NM_000535		1
PMS2	Exon 10 of 15 (deletion)	P	NM_000535.7		1
PMS2	Exon 10 of 15 (deletion)	P	NM_000535		10
PMS2	Exon 3-10 of 15 (Duplication)	LP	NM_000535		1
PMS2	Exon 3-7 of 15 (deletion)	P	NM_000535.7		1
PMS2	Exon 3-7 of 15 (deletion)	P	NM_000535		1
PMS2	Exon 5-9 of 15 (deletion)	P	NM_000535		2
PMS2	Exon 6-15 of 15 (deletion)	LP	NM_000535.7		1
PMS2	Exon 6-8 of 15 (deletion)	P	NM_000535		1
PMS2	Exon 7-10 of 15 (deletion)	LP	NM_000535		1
PMS2	Exon 7-10 of 15 deletion	LP	NM_000535		1
PMS2	Exon 8 of 15 (deletion)	P	NM_000535		1
PMS2	Exon 9-10 of 15 (deletion)	LP	NM_000535		1

© 2026 Sonawane KA et al. *JAMA Network Open*.

eTable 3. Demographic Distribution, Cancer Incidence, and Hypercholesterolemia Prevalence in South Carolina Counties

County	Age group, *% population*		Race/Ethnicity, *% population*					Females, *% population*	Breast Cancer Incidence (*per 100,000*)	Ovarian Cancer Incidence (*per 100,000*)	Colorectal Cancer Incidence (*per 100,000*)	High Cholesterol *% population*
	18-64 yrs	*≥65 yrs*	*NH White*	*NH Black*	*American Asian*	*American Indians /Alaska Natives*	*Native Hawaiian /Pacific Islander*					
Abbeville	79.7	23.0	69.9	25.9	0.4	0.4	NR	51.3	99.6	NR	32.6	34.0
Aiken	78.3	21.1	65.1	25.1	1.2	0.6	0.1	51.6	110.2	8.4	33.0	31.2
Allendale	80.4	22.4	22.7	72.1	1.0	0.5	0.1	46.9	89.0	NR	34.3	33.0
Anderson	77.1	18.6	76.5	16.0	1.2	0.4	0.1	51.5	127.3	7.6	41.8	33.2
Bamberg	80.0	24	36.5	58.5	0.9	0.6	0.1	52.3	122.0	NR	43.5	32.8
Barnwell	76.1	19.7	51.1	43.1	0.9	0.7	NR	51.5	107.1	NR	38.9	33.5
Beaufort	81.5	28.9	69.4	16.7	1.5	0.4	0.1	50.6	122.1	7.9	29.6	31.9
Berkeley	76.1	15.2	62.3	24.8	2.7	0.7	0.1	50.2	120.1	7.5	31.5	33.5
Calhoun	80.8	24.8	54.7	39.3	0.5	0.7	0.1	51.7	135.3	NR	49.8	31.9
Charleston	80.3	18.2	66.7	24.3	2.0	0.4	0.1	51.5	136.9	7.9	30.5	32.2
Cherokee	76.9	17.6	71.8	20.3	0.7	0.6	0.2	50.6	125.0	NR	46.0	33.8
Chester	77.4	19.7	58.9	35.6	0.6	0.6	0.1	51.6	138.8	NR	34.5	34.0
Chesterfield	77.8	19.6	58.9	32.5	0.7	0.9	0.1	50.9	133.6	NR	41.9	34.1

© 2026 Sonawane KA et al. *JAMA Network Open*.

Clarendon	80.7	25.1	49.8	45.2	0.8	0.6	NR	50.7	147.1	NR	44.5	31.8
Colleton	77.5	20.6	57.6	35.4	0.5	1.0	0.1	51.5	115.2	NR	41.8	31.2
Darlington	77.6	19.9	53.8	41.6	0.6	0.5	0.1	52.5	106.3	NR	51.9	33.5
Dillon	74.6	18.0	44.9	46.5	0.6	3.2	NR	52.9	125.6	NR	51.9	33.4
Dorchester	75.5	15.4	62.3	26.2	2.3	0.7	0.2	51.2	126.9	9.1	35.8	31.7
Edgefield	82.1	20.2	57.7	32.9	0.7	0.6	0.1	45.9	133.1	NR	29.3	32.8
Fairfield	81.2	24.3	39.6	55.0	0.8	0.5	NR	51.7	119.5	NR	40.5	34.2
Florence	76.2	18.1	45.8	47.5	2.6	0.6	0.1	53.0	134.3	9.2	39.2	34.7
Georgetown	82.0	31.0	66.7	28.6	0.7	0.4	0.1	52.6	141.0	12.6	44.5	29.1
Greenville	76.8	16.9	67.2	18.2	2.9	0.5	0.1	51.3	132.6	7.8	31.9	32.2
Greenwood	77.2	19.7	59.1	18.2	1.3	0.6	0.1	53.1	122.5	9.2	40.2	31.7
Hampton	78.3	20.0	41.2	52.2	0.7	0.4	NR	47.7	130.5	NR	52.8	34.0
Horry	82.1	26.6	77.7	12.3	1.5	0.6	0.1	51.6	114.3	10.3	35.2	34.5
Jasper	80.1	24.0	48.1	35.8	0.8	1.1	0.1	49.7	117.6	NR	30.2	33.7
Kershaw	76.7	19.2	66.8	25.0	0.9	0.5	0.1	51.4	119.7	8.0	31.1	34.3
Lancaster	78.3	21.8	68.7	20.3	2.7	0.4	0.1	50.9	128.9	6.6	32.1	34.0
Laurens	77.9	19.2	67.3	24.6	0.7	0.5	0.2	51.3	116.3	7.4	43.3	33.3
Lee	80.7	20.8	33.6	62.0	0.5	0.5	0.1	49.7	120.6	NR	39.5	33.3
Lexington	76.8	17.3	72.3	16.7	2.5	0.6	0.1	51.2	131.8	8.8	33.8	32.7

© 2026 Sonawane KA et al. *JAMA Network Open*.

Marion	77.2	21.7	38.0	56.2	0.7	0.9	NR	53.2	117.7	NR	53.7	32.2
Marlboro	79.8	19.4	38.9	50.6	0.4	5.0	0.1	47.3	122.7	NR	40.9	34.8
McCormick	88.4	38.8	56.6	39.0	0.7	0.3	0.1	47.0	140.2	NR	43.1	34.2
Newberry	78.2	20.8	60.6	29.3	0.7	0.9	0.3	50.7	140.3	NR	42.1	33.6
Oconee	80.1	24.8	84.0	7.5	0.8	0.4	NR	50.6	126.1	10.2	37.7	33.3
Orangeburg	77.9	21.1	33.3	61.6	0.1	0.7	0.1	52.9	120.4	12.1	50.7	33.1
Pickens	81.0	17.4	84.6	7.2	2.0	0.3	NR	50.0	134.8	9.5	36.5	32.5
Richland	78.4	14.2	40.4	49.4	3.1	0.4	0.1	51.9	147.3	8.1	30.7	32.8
Saluda	78.1	21.0	59.1	24.0	0.5	1.6	0.9	48.8	134.6	NR	35.7	34.2
Spartanburg	76.6	16.5	66.8	20.7	2.7	0.4	0.1	51.2	126.7	6.9	35.7	34.3
Sumter	75.8	17.8	44.4	47.9	1.4	0.5	0.1	51.7	116.8	8.6	36.4	32.8
Union	78.5	21.6	64.4	30.9	0.5	0.4	0.1	52.5	152.4	NR	44.3	34.5
Williamsburg	79.2	22.9	32.4	62.9	0.8	0.6	NR	51.1	136.2	NR	46.0	32.8
York	75.7	15.5	68.0	19.4	3.5	0.8	0.1	51.3	140.3	8.4	32.9	31.7

Abbreviations: NH, Non-Hispanic; NR, Not reported.

© 2026 Sonawane KA et al. *JAMA Network Open*.

eTable 4. Urban-Rural Continuum (RUC) Classification, Social Vulnerability, and Population Sizes of South Carolina Counties

County	RUC code†	Social Vulnerability‡					Population
		Overall	Household Characteristics	Housing and Transportation	Minority Status	Socioeconomic Status	Overall (18 years and older)
Abbeville	6	0.27	0.36	0.04	0.16	0.47	19,572
Aiken	2	0.22	0.18	0.38	0.36	0.13	134,885
Allendale	8	1	1	0.87	1	0.93	6,291
Anderson	2	0.18	0.2	0.13	0.07	0.24	160,100
Bamberg	9	0.89	0.98	0.78	0.91	0.47	10,720
Barnwell	8	0.84	0.78	0.93	0.69	0.87	15,699
Beaufort	3	0.07	0.02	0.22	0.2	0.22	157,927
Berkeley	2	0.2	0.09	0.42	0.42	0.16	181,906
Calhoun	2	0.42	0.4	0.31	0.6	0.36	11,498
Charleston	2	0.13	0.16	0.02	0.33	0.31	333,213
Cherokee	6	0.44	0.56	0.33	0.11	0.47	43,372
Chester	1	0.38	0.56	0.09	0.53	0.29	24,965
Chesterfield	6	0.8	0.71	0.98	0.47	0.84	33,912
Clarendon	8	0.87	0.82	0.44	0.71	1	25,281
Colleton	6	0.82	0.87	0.71	0.58	0.91	29,860

© 2026 Sonawane KA et al. *JAMA Network Open*.

Darlington	3	0.62	0.6	0.69	0.62	0.64	48,702
Dillon	6	0.96	0.96	0.96	0.76	0.78	20,907
Dorchester	2	0.31	0.33	0.56	0.4	0.2	124,573
Edgefield	2	0.56	0.49	0.58	0.56	0.73	21,935
Fairfield	2	0.67	0.62	0.62	0.84	0.69	16,922
Florence	3	0.49	0.38	0.27	0.67	0.73	104,482
Georgetown	4	0.11	0.24	0.07	0.31	0.09	53,101
Greenville	2	0.24	0.13	0.29	0.24	0.38	413,455
Greenwood	4	0.51	0.42	0.64	0.49	0.42	53,702
Hampton	8	0.91	0.89	1	0.82	0.64	14,722
Horry	2	0.36	0.53	0.51	0.04	0.11	305,319
Jasper	3	0.69	0.64	0.51	0.73	0.82	24,820
Kershaw	2	0.02	0.11	0.4	0.22	0	51,367
Lancaster	1	0.04	0.04	0.49	0.13	0.04	79,098
Laurens	2	0.64	0.69	0.82	0.27	0.58	52,957
Lee	8	0.93	0.93	0.2	0.96	0.98	13,251
Lexington	2	0.09	0.07	0.36	0.09	0.18	230,910
Marion	6	0.73	0.73	0.73	0.89	0.71	22,406
Marlboro	6	0.76	0.91	0.16	0.87	0.89	21,007

© 2026 Sonawane KA et al. *JAMA Network Open*.

McCormick	8	0.29	0.22	0.16	0.64	0.27	8,687
Newberry	6	0.47	0.27	0.91	0.44	0.4	29,847
Oconee	4	0.33	0.44	0.76	0.02	0.07	64,164
Orangeburg	4	0.78	0.84	0.6	0.93	0.8	65,477
Pickens	2	0.16	0.29	0	0	0.6	107,754
Richland	2	0.53	0.47	0.22	0.8	0.62	327,912
Saluda	2	0.58	0.51	0.89	0.51	0.44	14,812
Spartanburg	2	0.4	0.31	0.64	0.29	0.33	258,968
Sumter	3	0.6	0.64	0.47	0.78	0.53	79,798
Union	2	0.71	0.8	0.84	0.38	0.56	21,304
Williamsburg	6	0.98	0.76	0.8	0.98	0.96	24,513
York	1	0	0	0.11	0.16	0.02	219,639

Abbreviations: RUC, Rural-Urban Continuum

†Rural-Urban Continuum codes classify counties into nine categories, from 1 (most urban; yellow) to 9 (most rural; dark green) based on metro population size, degree of urbanization, and proximity to metropolitan areas. They distinguish metropolitan counties by the size of their metro area and nonmetropolitan counties by how urbanized they are and whether they are adjacent to a metro area.

‡Social Vulnerability Index measures how well communities can prepare for, respond to, and recover from disasters by assessing social and demographic factors. It combines multiple characteristics into four component themes: socioeconomic status (e.g., poverty, unemployment, income, education), household composition & disability (e.g., age, disability, single-parent households), minority status & language (e.g., race/ethnicity, English proficiency), and housing type & transportation (e.g., crowded housing, access to vehicles). Each community receives a score from 0 (least vulnerable; dark blue) to 1 (most vulnerable; dark red), helping identify areas needing targeted support.

© 2026 Sonawane KA et al. *JAMA Network Open*.

eTable 5. Population Sizes of South Carolina Counties by Urban-Rural Continuum (RUC) Classification

RUC code[†]	No. of counties	Population (18 yrs and older)
1	3	168,531
2	18	2,769,790
3	5	415,729
4	4	236,444
5	None	-
6	9	245,396
7	None	-
8	6	83,931
9	1	10,720

© 2026 Sonawane KA et al. *JAMA Network Open*.

eTable 6. Population Sizes of South Carolina by County Social Disadvantage

Social Vulnerability†	No. of counties	Population (18 yrs and older)
Overall		
Quartile 1	12	2,123,355
Quartile 2	11	995,447
Quartile 3	11	685,270
Quartile 4	12	281,640
Household Characteristics		
Quartile 1	12	2,024,288
Quartile 2	11	1,124,407
Quartile 3	11	667,985
Quartile 4	12	269,032
Housing and Transportation		
Quartile 1	12	1,447,128
Quartile 2	11	1,356,052
Quartile 3	11	972,684
Quartile 4	12	309,848
Minority Status		
Quartile 1	12	1,852,677
Quartile 2	11	1,278,368
Quartile 3	11	330,741
Quartile 4	12	623,926
Socioeconomic Status		
Quartile 1	11	1,762,989
Quartile 2	11	1,222,811
Quartile 3	12	818,894
Quartile 4	12	281,018

© 2026 Sonawane KA et al. *JAMA Network Open*.

eTable 7. Logistic Regression Model Evaluating Withdrawal After Initial Consent, *In Our DNA SC*[*]

Variable[**]	Odds Ratio (95% Confidence Interval) [†]
**Rurality**	
Rural	Ref
Urban	1.26 (0.80-2.00)
**Social Vulnerability**	
Quartile 1	Ref
Quartile 2	0.76 (0.42-1.39)
Quartile 3	0.64 (0.36-1.17)
Quartile 4	0.90 (0.51-1.60)

[*]A total of 542 individuals had withdrawn (by July 2025) after initial consent to participate.
[**]Rurality modeled as a binary variable due to limited sample. Social vulnerability quartiles indicate lowest to highest vulnerability: Quartile 1 (0.00-0.25), Quartile 2 (0.51-0.75), Quartile 3 (0.26-0.50), and Quartile 4 (0.76-1.00).
[†]Logistic regression model was simultaneously adjusted for age, sex, race, ethnicity, rurality, and social vulnerability.

© 2026 Sonawane KA et al. *JAMA Network Open*.
