# Supplementary material for: Geographic and Social Equity in Population-Wide Genomic Screening
Source: JAMA Netw Open. 2026 Jul 13;9(7):e2622743. doi: 10.1001/jamanetworkopen.2026.22743 (PMC13366200; doi:10.1001/jamanetworkopen.2026.22743)
Supplement: Supplement 2. — Data Sharing Statement [file jamanetwopen-e2622743-s002.pdf]

Data Sharing Statement

Sonawane. Geographic and Social Equity in Population-Wide Genomic Screening. *JAMA Netw Open*. Published July 13, 2026. doi:10.1001/jamanetworkopen.2026.22743

Data
Data available: Yes
Data types: Deidentified participant data
How to access data: De-identified data is available after institutional approval and approval from MUSC IRB. Please send data requests to inourdnasc@musc.edu
When available: With publication

Supporting Documents
Document types: None

Additional Information
Who can access the data: Researchers whose proposed use of the data has been approved.
Types of analyses: Non-commercial research purposes only.
Mechanisms of data availability: De-identified data is available after institutional approval and approval from MUSC IRB after completing data access agreement.
